# Further In Vitro Assessment and Mid-Term Evaluation of Control Strategy of *Xylella fastidiosa* subsp. *pauca* in Olive Groves of Salento (Apulia, Italy)

**DOI:** 10.3390/pathogens10010085

**Published:** 2021-01-19

**Authors:** Giuseppe Tatulli, Vanessa Modesti, Nicoletta Pucci, Valeria Scala, Alessia L’Aurora, Simone Lucchesi, Manuel Salustri, Marco Scortichini, Stefania Loreti

**Affiliations:** 1 Council for Agricultural Research and Economics (CREA), Research Centre for Plant Protection and Certification, 00156 Roma, Italy; giuseppe.tatulli@crea.gov.it (G.T.); vanessamodesti@libero.it (V.M.); nicoletta.pucci@crea.gov.it (N.P.); valeria.scala@crea.gov.it (V.S.); alessia.laurora@crea.gov.it (A.L.); simone.lucchesi@crea.gov.it (S.L.); 2 Dipartimento di Biologia Ambientale, Sapienza University of Rome, 00185 Roma, Italy; manuel.salustri@uniroma1.it; 3 Council for Agricultural Research and Economics (CREA), Research Centre for Olive, Fruit and Citrus Crops, 00134 Roma, Italy

**Keywords:** olive quick decline syndrome, zinc, copper, real-time PCR, fertilizer

## Abstract

During recent years; *Xylella fastidiosa* subsp. *pauca* (Xfp) has spread in Salento causing relevant damage to the olive groves. Measures to contain the spreading of the pathogen include the monitoring of the areas bordering the so-called “infected” zone and the tree eradication in case of positive detection. In order to provide a control strategy aimed to maintain the tree productivity in the infected areas, we further evaluated the in vitro and *in planta* mid-term effectiveness of a zinc-copper-citric acid biocomplex. The compound showed an in vitro bactericidal activity and inhibited the biofilm formation in representative strains of *X. fastidiosa* subspecies, including Xfp isolated in Apulia from olive trees. The field mid-term evaluation of the control strategy assessed by quantitative real-time PCR in 41 trees of two olive groves of the “infected” area revealed a low concentration of Xfp over the seasons upon the regular spraying of the biocomplex over 3 or 4 consecutive years. In particular, the bacterial concentration lowered in July and October with respect to March, after six consecutive treatments. The trend was not affected by the cultivar and it was similar either in the Xfp-sensitive cultivars Ogliarola salentina and Cellina di Nardò or in the Xfp-resistant Leccino. Moreover, the scoring of the number of wilted twigs over the seasons confirmed the trend. The efficacy of the treatment in the management of olive groves subjected to a high pathogen pressure is highlighted by the yielded a good oil production

## 1. Introduction

In recent years, *Xylella fastidiosa* subsp. *pauca* (Xfp) has been found associated with the “olive quick decline syndrome” (OQDS) in the Salento area (Apulia region, southern Italy) [1,2,3]. Main symptoms include leaf, twig, and branch wilting, often followed by the plant death. After the first record in the Gallipoli area (Lecce province), in accordance with the European legislation that rules quarantine pests and pathogens, the spread and occurrence of this phytopathogen was surveyed and monitored by ad hoc authorities of Apulia Region [4]. So far, the bacterium has reached most of olive groves of Lecce province and some areas of Taranto, Brindisi and Bari provinces. A recent study based on a satellite data monitoring on the occurrence of OQDS in these areas estimates that about 6,500,000 olive trees are affected by the disease [5].

In Apulia, Xfp can survive also in some wild plant species other than olive [6], and it is mainly spread within and between olive groves by the common meadow spittlebug *Philaenus spumarius* that can expand the pathogen up to 20 km per year [7]. Due to the particular topology of such olive orchards that persist over many kilometres in the territory, it has been retained that Xfp will persist in those areas in the following years [8] with possibilities to reach also other neighbouring regions in the future causing very severe economic losses to the oil industry of southern Italy [9]. Nowadays, in some parts of the Lecce province, whole areas previously cultivated with olive show a total collapse of trees, whereas some others are characterized by an extensive tree decline.

Measures to contain the further spreading of the pathogen include the monitoring of the areas bordering the so-called “infected” area that include the whole Lecce province and part of Taranto and Brindisi provinces. The surveys have the aim to possibly detect Xfp in both symptomatic and asymptomatic olive trees within the “containment” and “buffer” zones that are located north of the “infected” one. Upon the laboratory assays, trees that result positive in the “containment” area must be uprooted in accordance with the current European phytosanitary legislation, whereas if a positive case is detected within the “buffer” area also all the olive trees and the specified plants present in a 50 m radius from the infected, one must be uprooted.

Besides the control of vector(s) through insecticides, other proposed solutions to mitigate the impact of Xfp in Salento include the planting or grafting of resistant cultivars such as Leccino [10] or Fs17^®^ [11] in place of the sensitive local cultivars Ogliarola salentina and Cellina di Nardò [12]. The substitution of the olive groves with other fruit tree crops such as mango and avocado has been also proposed [13]. Previously, we have ascertained the in vitro antibacterial activity of an internationally patented biocomplex that contains zinc (4%), copper (2%) and citric acid, namely, Dentamet^®^, towards one strain of *X. fastidiosa* subsp. *fastidiosa*, evaluating its field effectiveness to control Xfp in an olive orchard of Salento [14]. In the study, we also showed that zinc and copper reached the xylem network of the treated trees and that such ions are released in the xylem vessels but are not present in the oil. The field trial regarded the local cultivars Ogliarola salentina and Cellina di Nardò that yield an oil among the richest in the polyphenol content [15,16]. Such cultivars also represent an invaluable heritage in terms of history, landscape and social traditions [17]. The control approach we tested was adopted by some farmers of the “infected” area, which start to apply it as a routine measure in their olive groves to face the Xfp epidemic. Since the mid and long-term effectiveness of a control strategy is a basic pre-requisite for its wider application in a territory, and the efficacy of the product “has to be assessed under conditions as near as possible to the conditions of practical use of the product” [18], it is important to verify the robustness of the protocol in farms in production. In order to evaluate the practical application of the control strategy in the mid-term period, we thoroughly assessed both the field symptoms and Xfp DNA concentrations on two olive groves of the “infected” area that regularly applied the biocomplex over three and four consecutive years, respectively, taking into account three different cultivars typical of the Salento areas (Leccino, Ogliarola salentina and Cellina di Nardò). This assessment does not represent an experimental study aimed to assess the effectiveness of the treatments as previously investigated [14], but rather an evaluation of a state of the situation in olive groves that applied this control strategies over 3 or 4 years, as a routine agronomical technique. Finally, this study was also aimed to further investigate the in vitro antibacterial activity of Dentamet^®^ towards *X. fastidiosa* by including also representative strains of the subspecies *fastidiosa* and *multiplex* as well as of the subsp. *pauca* isolated in Apulia from olive trees.

## 2. Results

### 2.1. In Vitro Antibacterial Activity, Agar Dilution and Biofilm Inhibition Assays

**Figure 1:**
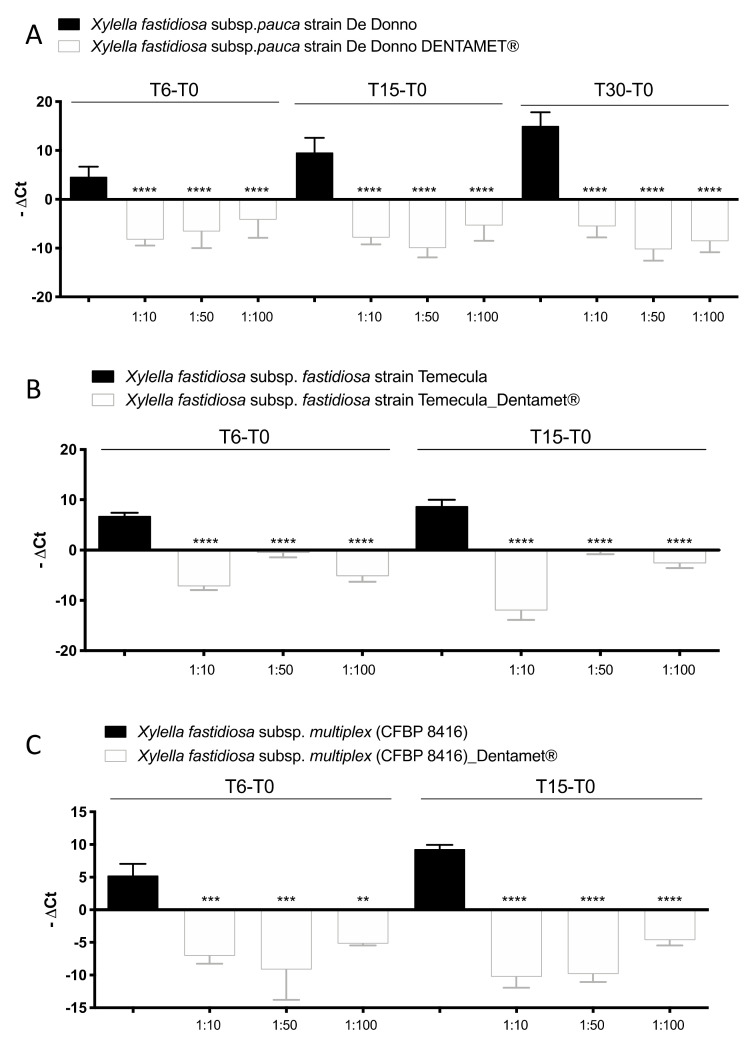
*Xylella fastidiosa* subsp. *pauca* strain De Donno (CFBP8402), *X. fastidiosa* subsp. *fastidiosa* (Xff) strain Temecula and *X. fastidiosa* subsp. *multiplex* (Xfm) (CFBP 8416) grown in addition with 1:10, 1:50 and 1:100 Dentamet^®^ dilutions and without Dentamet^®^ (control). Planktonic growth was assessed by real-time PCR on gDNA (**A**–**C**). −ΔCt represents the difference between the Ct value obtained at each time point (respectively 6, 15, 30 dpi depending of *X. fastidiosa* subspecies) and the Ct value at time 0 for each sample. Values are means ± SD of the most representative experiment of two independent experiments (*n* = 8 in total). A statistically significant difference was obtained between controls Xfp, Xff and Xfm (grown in PD2 broth) and the same strain added with Dentamet^®^ dilutions, according to one-way ANOVA, Dunnett’s test (** *p* ≤ 0.002; *** *p* ≤ 0.0003; **** *p* ≤ 0.0001 vs. Ctr).

Preliminary experiments were set up on Xfp strain De Donno with a large range of Dentamet^®^ dilutions to detect the minimum inhibition concentration (MIC). Dilution greater than 1:100 did not exert any inhibition; therefore, the experiments were focused on Dentamet^®^ dilutions of 1:10, 1:50 and 1:100. The real-time PCR results on bacterial suspension revealed an inhibition of bacterial growth from 1:10 to 1:100 Dentamet^®^ dilution (Supplemental Figure S1). No curve amplification was obtained from tubes containing Dentamet^®^ diluted at 1:10 and 1:50 by processing broth bacterial cultures by real-time PCR (Supplemental Figure S1), in addition, the inhibition effect of Dentamet^®^ was confirmed at 1:100 dilution, as the bacterial growth was reduced with respect the initial bacterial concentration (Supplemental Figure S1). As the absence of curve amplification at lower Dentamet^®^ dilutions (1:10 and 1:50) could be due to a possible interference of Dentamet^®^, the same Dentamet^®^ dilutions were assessed by real-time PCR of gDNA extracted from broth culture of *X. fastidiosa* subspecies. Its antibacterial activity towards Xfp, Xff and Xfm was evaluated by assessing: (i) the planktonic growth and biofilm production in PD2 broth and (ii) the bacteriostatic/bactericide Dentamet^®^ efficacy by plating aliquots of decimal dilution from 10^7^–10^3^ CFU mL^−1^ of *X. fastidiosa* inoculum on PD2 agar plates. The result of the planktonic growth was evaluated by real-time PCR of gDNA extracted from each bacterial culture at time 0 and after 6, 15 days (Xfm and Xff) and 30 days post treatment (Xfp) (Figure 1).

Finally, at the end of the trials, an aliquot of each experimental bacterial growth was plated on PD2 agar. No growth was observed for all the Dentamet^®^ dilutions, including the 1:100; whereas the growth of typical *X. fastidiosa* colonies were observed in the positive control (data not shown). These findings indicate that the MBC of the biocomplex is 1:100, corresponding at 400 mg L^−1^ (2.5 mM) for zinc and 200 mg L^−1^ (1.2 mM) for copper.

After 6 days of treatment there was a significative gDNA decrease of all *X. fastidiosa* subspecies in all Dentamet^®^ dilutions, with respect to the Xf controls, and this trend was confirmed also at 15 (Xfp, Xff, Xfm) and 30 days post the treatment indicating a long last effect (Xfp). It is worth of mention that in the untreated control the Ct decreased constantly up to 15 and 30 days, confirming the adequate increment of Xf gDNA extracted from the bacterial culture grown during the experiment (Figure 1). Furthermore, it should be noted that in each Dentamet^®^ dilution, the concentration of gDNA of all Xf was lower than those inoculated and detected at T0 (−ΔCt negative values). These results support that Dentamet^®^ has an antibacterial (bactericidal or bacteriostatic) activity towards Xfp, Xff and Xfm up to a dilution of 1:100. The bactericidal effect of 1:100 dilution was confirmed for the three tested strains of *X. fastidiosa* by plating on PD2 agar an aliquot of bacterial suspension at the end of each trial. After 30 days no growth was observed for all subspecies (data not shown).

**Figure 2:**
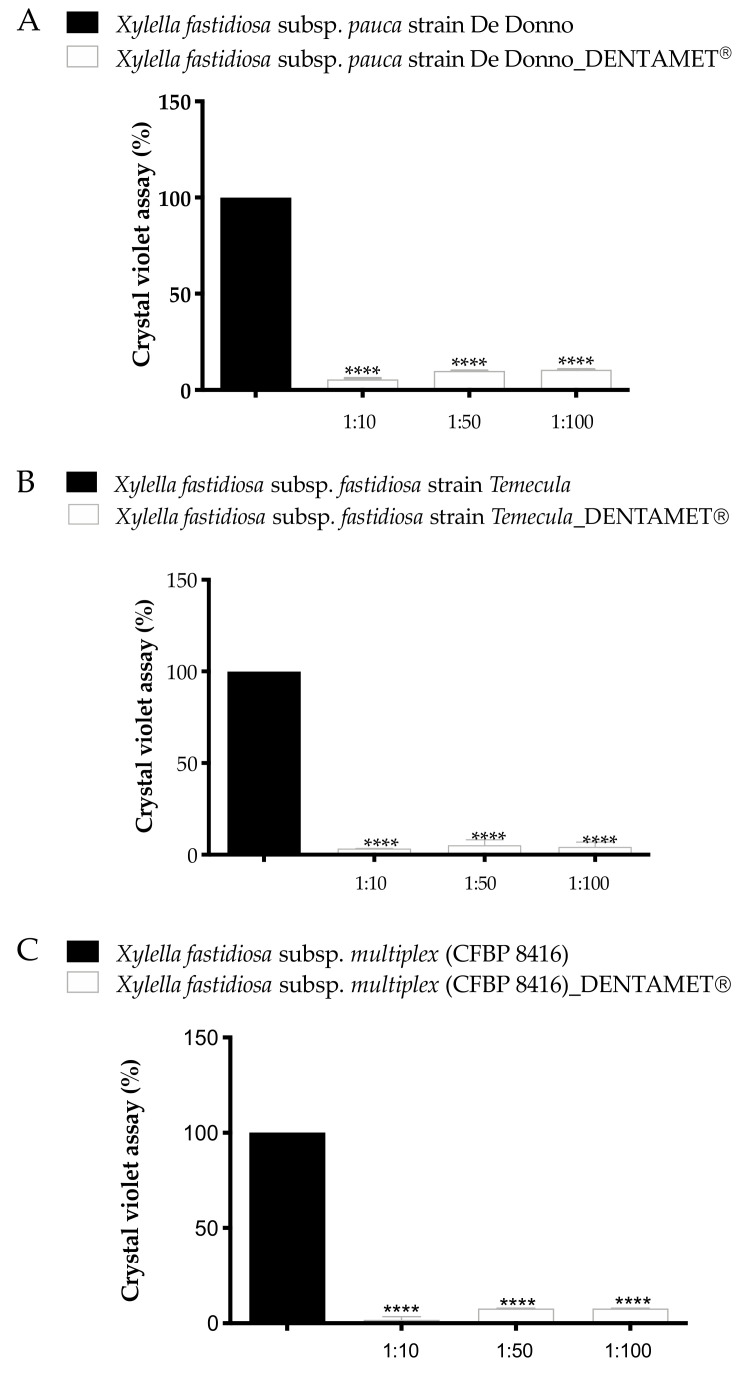
Biofilm assay 30 dpi of *X. fastidiosa* subsp. *pauca* (De Donno_CFBP8402) (Xfp) (**A**), and 15 dpi of *X. fastidiosa* subsp. *fastidiosa* (Temecula1) (Xff) (**B**) and *X.fastidiosa* subsp. *multiplex* (CFBP 8416) (Xfm) (**C**). The ordinate axes reports % of biofilm formation, the abscissa axes the Dentamet^®^ dilutions. Values are means ± SD of three independent biological replicates (*n* = 7). A statistically significant difference was obtained between Xfp, Xff and Xfm PD2 cultures (untreated control) and the same subspecies added with Dentamet^®^ dilutions (normalized with absorbance obtained by tubes without bacteria), according to one-way ANOVA, Dunnett’s test (**** *p* ≤ 0.0001 vs. Ctr).

The biofilm quantification assay reported in Figure 2 showed that untreated controls produce biofilm in an average range of OD600nm between 1 and 2.5 (Xfm 0.9 OD, Xff 1.4 OD, Xfp 2.5 OD), while the bacteria cell culture treated with all Dentamet^®^ dilutions significantly reduce the biofilm production in all strains of *X. fastidiosa* subspecies (Supplemental Figure S2). The higher biofilm production of Xfp De Donno strain with respect Xff Temecula1, confirm the evidence that De Donno strain has a more aggregative phenotype than Temecula1 [19].

**Figure 3:**
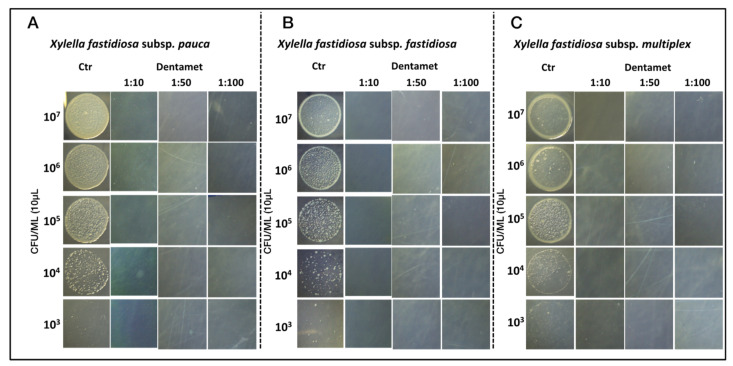
Bacteriostatic/bactericidal evaluation of *X. fastidiosa* subsp. *pauca* strain De Donno_CFBP8402 (**A**), *X*. *fastidiosa* subsp. *fastidiosa* strain Temecula1 (**B**) and *X*. *fastidiosa* subsp. *multiplex* strain CFBP 8416 (**C**). Aliquots of 10 µL and ten-fold dilutions from 10^7^ to 10^3^ CFU mL^−1^ of each *X*. *fastidiosa* subspecies were spotted on PD2 agar plates supplemented or not with 1:10, 1:50 and 1:100 Dentamet^®^ dilutions and incubated at 28 °C. Ctr: *X*. *fastidiosa* cultures grown on PD2 Dentamet^®^-free.

Finally, in order to corroborate the bactericidal efficacy of the biocomplex observed in Figure 1 and Figure 2, the growth inhibition of the three strains of *X. fastidiosa* subspecies (Xfp, Xff and Xfm) was assessed in plates supplemented with all Dentamet^®^ dilutions. The results (showed in Figure 3A–C) highlight the absence of growth of the three *X. fastidiosa* strains on PD2 added with Dentamet^®^ dilutions, in comparison with the growth on PD2 Dentamet^®^ -free.

### 2.2. Field Symptoms Assessment and Tree Yield

The assessment of Dentamet^®^ application was tested on two olive groves of the “infected” Salento areas, Cannole and Galatone, on Leccino, Ogliarola salentina and Cellina di Nardò at Galatone and on Ogliarola salentina and Cellina di Nardò at Cannole. Plant field symptoms, olive production and Xfp equivalents per g of leaf (i.e., CFU g^−1^) were evaluated.

**Figure 4:**
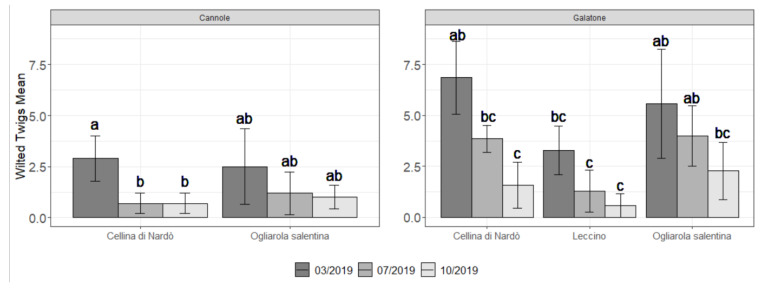
Mean number ± standard error of new wilted twigs on olive trees treated with Dentamet^®^ as assessed on March, July and October 2019 at Cannole (Ogliarola salentina, Cellina di Nardò) and Galatone (Ogliarola salentina, Cellina di Nardò, Leccino) (Lecce province). Statistical significance was assessed through Multiple ANOVA (*p*-value < 0.05) after having testes the data normality, followed by post-hoc Tukey’s test (alpha = 0.05). Different letters indicate statistical difference between group means according to the Tukey’s test.

The number of new wilted twigs as recorded in both plots during 2019 is shown in Figure 4.

**Figure 5:**
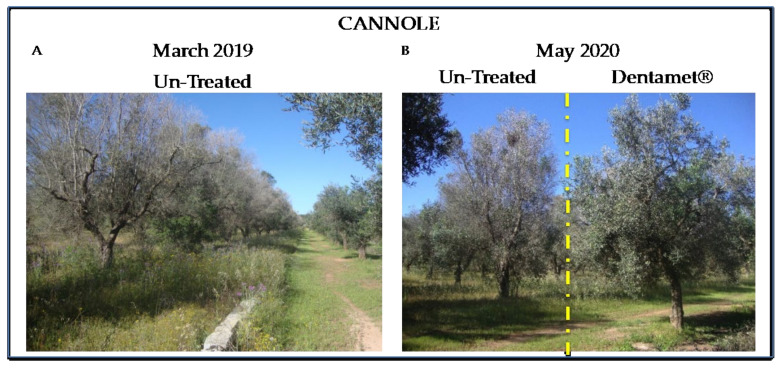
Cannole plot trees. (**A**) Control untreated trees in Cannole plot as observed in March 2019. The trees appeared severely damaged by *X. fastidiosa* subsp. *pauca*. (**B**) Ogliarola salentina trees treated with Dentamet^®^ (right) and control tree (left) on May 2020, one year after the data samplings.

**Figure 6:**
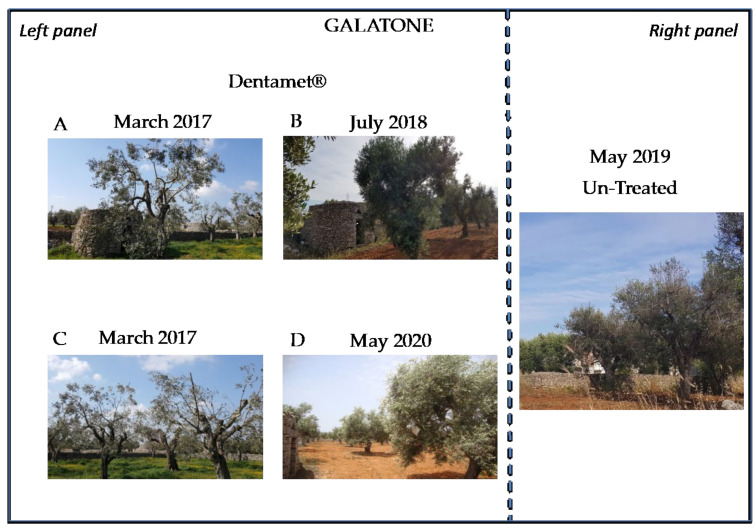
Galatone plot trees. (**Left panel**) (**A**) Olive trees of Galatone Plot as appeared in March 2017 just before the starting of the treatments; (**B**) the same trees in July 2018, 17 months after the starting of the spray treatments with Dentamet^®^. (**C**) Ogliarola salentina and Cellina di Nardò trees in Galatone plot as appeared in March 2017 just before the starting of spray treatments; (**D**) Leccino (foreground) and Cellina di Nardò (background) trees of Galatone plot in May 2020, one year after the present study. (**Right panel**) Control trees in Galatone plot as observed in March 2019. The trees appeared severely damaged by *X. fastidiosa* subsp. *pauca*.

A trend that indicates a reduction of the field symptoms during the year in both plots and for all cultivars was observed. Wilted twigs, indeed, are higher in March and decrease in July and October. Ogliarola salentina and Cellina di Nardò cultivars were confirmed more sensitive to Xfp in comparison with Leccino as observed in Galatone. In both plots, Cellina di Nardò showed in March a statistically significant higher number of wilted twigs when compared with Ogliarola salentina and Leccino at Cannole, and with Leccino at Galatone. However, in any case at the end of the season, just before the harvest time, few new wilted twigs per tree were recorded for all cultivars in both plots (i.e., a maximum of 1-2 for the Ogliarola salentina trees) (Figure 4). This feature was also confirmed by the visual assessment of the part of the farm not subjected to the assessment (Figure 5 and Figure 6).

**Figure 7:**
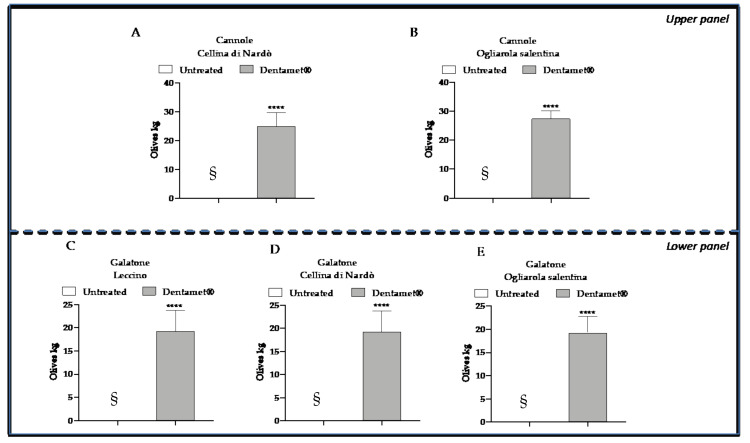
Plant production. Graphical representation of olives production of (**A**) Cellina di Nardò and (**B**) Ogliarola salentina at Cannole (upper panel) and (**C**) Leccino, (**D**) Cellina di Nardò and (**E**) Ogliarola salentina in Galatone (Lower panel) expressed as means ± SD of Kg olives per plant. § Un-treated control plants were completely withered during 2019 in Galatone and in Cannole and determined no olives production. A statistically significant difference was obtained between un-treated plants and plants treated with Dentamet^®^, according to one-sample T-test and Wilcoxon test (**** *p* ≤ 0.0001 vs. controls (zero olives Kg)).

The healthy status of the treated trees is confirmed by their yield that shows a satisfactory production, estimated at about 18–19 and 22–23 Kg of olive per tree, as recorded in autumn 2019 at Galatone and Cannole, respectively. Un-treated trees resulted completely wilted (Figure 7).

### 2.3. Quantitative Real-Time PCR Assessment of Xfp within Olive Leaves

**Figure 8:**
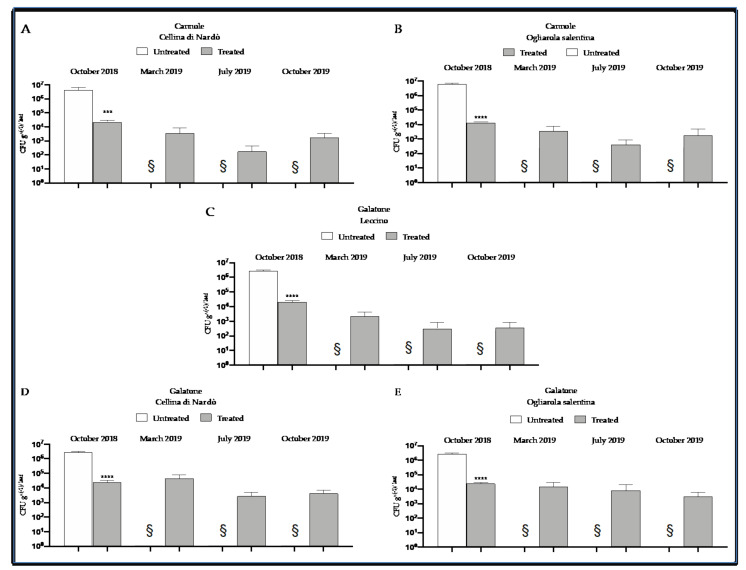
*X. fastidiosa* subsp. *pauca* DNA concentration, expressed in CFU equivalents g^−1^ of leaf, determined for un-treated plant (assessed as time 0 control) and Dentamet^®^-treated cultivars Cellina di Nardò, Ogliarola salentina, at Cannole and Galatone (assessed in 2019). § Untreated control plants dead in 2019, so the absence of concentration data is indicative of plant death. (**A**,**B**) Graphical representation of the bacterial concentration over time of Cellina di Nardò and Ogliarola salentina at Cannole. (**C**–**E**) Graphical representation of the bacterial concentration over time of Leccino, Cellina di Nardò and Ogliarola salentina at Galatone. Bacterial concentration is expressed in CFU equivalents g^−1^ of leaf ((**** *p* ≤ 0.0001 vs. controls (zero olives Kg), see also Section 4).

In early March 2016, the following mean Xfp CFU equivalents g^−1^ of leaf were found at Cannole plot: 6.5 × 10^5^ ± 2.1 × 10^4^ SD and 5.7 × 10^5^ ± 1.8 × 10^4^ SD for Ogliarola salentina and Cellina di Nardò, respectively. In early March 2017, the following mean Xfp CFU equivalents g^−1^ of leaf were found at Galatone plot: 1.5 × 10^5^ ± 1.4 × 10^4^ SD (Leccino), 1.2 × 10^6^ ± 1.1 × 10^4^ SD (Ogliarola salentina), and 1.8 × 10^6^ ± 1.8 × 10^4^ SD (Cellina di Nardò). Subsequently, in both plots the trees were pruned to remove the withered twigs (see also Figure 6, left panel). In October 2018, un-treated and Dentamet^®^ treated trees of tipical Salento cultivars, in Cannole and Galatone plots, were chosen and assessed for time 0 control and Xfp concentration evaluation in CFU equivalents g^−1^ of leaf tissue. For the one-year evaluation of 2019, CFU equivalents g^−1^ of leaf tissue were assessed in treated plants in March, July and October 2019. Non-treated control plants were not assessed, as died in 2019 (see Section 4 (Figure 8).

Significant differences about bacterial DNA concentration and wilted twigs level were assessed, after verifying hypothesis of normality and equal variance, through Multiple ANOA (*p*-value < 0.0001) followed by post-hoc Tukey’s test (alpha = 0.05).

The mean bacterial concentration found in samples of Ogliarola salentina, Leccino and Cellina di Nardò (Galatone) and Ogliarola salentina and Cellina di Nardò (Cannole) leaves collected in the different months and in the two plots (Cannole and Galatone) is reported in Table 1.

**Figure 9:**
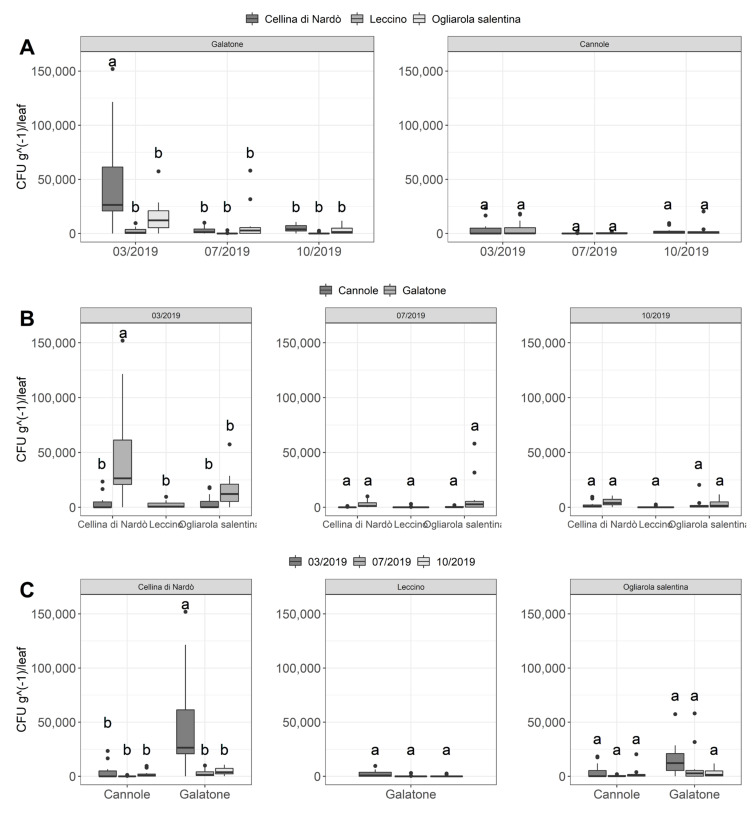
*X. fastidiosa* subsp. *pauca* DNA concentration, expressed in CFU g^−1^ of leaf, determined for Dentamet^®^-treated cultivars Cellina di Nardò, Leccino, Ogliarola salentina, at Galatone and in Ogliarola salentina and Cellina di Nardò at Cannole olive groves. The differences in CFU equivalents among the cultivars at different sampling data in the two orchards are reported. (**A**) Graphical representation of bacterial concentration for all cultivars in the different sampling periods. (**B**) Graphical representation of the bacterial concentration of Cellina di Nardò, Leccino, Ogliarola salentina, as revealed at Cannole and at Galatone. (**C**) Graphical representation of the variation over time of bacterial concentration between Cannole and Galatone of the Cellina di Nardò, Leccino, Ogliarola salentina. The bacterial concentration is expressed in CFU equivalents g^−1^ of leaf (see also Material and Methods). Statistical significance was assessed through Multiple ANOVA (*p*-value < 0.05) followed by post-hoc Tukey’s test (alpha = 0.05). Different letters indicate statistical difference between group means according to the Tukey’s test.

The overall data showed a low concentration of bacterial load for all cultivars in the different sampling periods that not differed statistically (Figure 9). An exception was the Cellina di Nardò sampling collected in March at Galatone (Figure 9A) that showed a higher statistically significant bacterial concentration (in the range of 4.5 × 10^4^ ± 4.4 × 10^4^ SD CFU g^−1^) with respect the Ogliarola salentina (1.5 × 10^4^ ± 1.5 × 10^4^ SD CFU g^−1^) and in particular Leccino (2.2 × 10^3^ ± 3.1 × 10^3^ SD CFU g^−1^) (Table 1). Considering the Galatone plot in March, the adjusted *p*-values from Tukey’s test for Cellina-Ogliarola and Cellina-Leccino comparisons are, respectively, 5.61 × 10^−8^ and 1.70 × 10^−13^. However, Cellina di Nardò showed a very high standard deviation that highlighted a higher difference among the concentrations of the collected samples in March with respect the other samplings.

Indeed, the Cellina di Nardò trees sampled in March at Cannole showed a very similar bacterial concentration of Ogliarola salentina (Figure 9B), estimated: 3.6 × 10^3^ ± 6.2 × 10^3^ SD and 3.7 × 10^3^ ± 5.9 × 10^3^ SD CFU g^−1^ of leaf in Cellina di Nardò and Ogliarola salentina, respectively (Table 1). The *p*-value from Tukey’s test for this comparison is, in fact, equal to 1, denoting a non-significant difference.

For all cultivars and both plots, the bacterial concentration decreased in July and in October following the application of the treatments (the total mean in March was 1.2 × 10^4^ ± 2.5 × 10^4^ SD with respect 2.1/2.2 × 10^3^ ± 7.4/3.4 × 10^3^ SD CFU g^-1^, respectively, in July and October (Table 1), and remained at low levels, not statistically different each other (Figure 9B, Table 1). This was evident also for Cellina di Nardò samples collected at Galatone, whose bacterial concentration did not statistically differ with the other cultivars in July and October (Figure 9C) (bacterial concentrations over the period July/October for Cellina di Nardò: 4.4 × 10^2^/4.4 × 10^3^ ± 5.9 × 10^2^/3.3 × 10^3^ SD CFU g^−1^, *p*-value = 1; for Ogliarola salentina: 8.3/3.1 × 10^3^ ± 1.6 × 10^4^/3.7 × 10^3^ SD CFU g^−1^ *p*-value = 1, and for Leccino 1.8/3.5 × 10^2^ ± 3.5/7.9 × 10^2^ SD CFU g^−1^, *p*-value = 1). The overall results also highlighted a higher pathogen inoculum in Galatone with respect to Cannole (Figure 9C). Taking into account all samplings and only the Xfp-sensitive cultivars Cellina di Nardò and Ogliarola salentina, the mean bacterial concentration found in Galatone was of about 9.0 × 10^3^ ± 2.1 × 10^4^ SD CFU g^−1^, whereas in Cannole was 1.9 × 10^3^ ± 4.2 × 10^3^ SD CFU g^−1^ (Table 1).

Finally, the comparison of the three cultivars located at Galatone, indicated that Leccino showed a lower bacterial inoculum (mean of about 9 × 10^2^ ± 2.1 × 10^3^ SD CFU g^−1^) with respect to Cellina di Nardò (1.7 × 10^4^ ± 3.2 × 10^4^ SD CFU g^−1^) and Ogliarola salentina (8.7 × 10^3^ ± 1.4 × 10^4^ SD CFU g^−1^) (Table 1, Figure 9). In this latter case, the adjusted *p*-values from Tukey’s test for Leccino-Ogliarola and Leccino-Cellina comparisons are, respectively, 4.2 × 10^−2^ and 4.13 × 10^−8^.

## 3. Discussion

To further investigate the in vitro efficacy of Dentamet^®^ and corroborate its antibacterial activity towards *X. fastidiosa*, in this study were tested representative strains of different subspecies of the pathogen respect to Scortichini et al., 2018 [14]. We observed a bactericidal activity of the compound from 1:10 to 1:100 towards all the three strains tested and representing *X. fastidiosa* subspecies [20,21], including a Xfp strain isolated from an olive tree showing OQDS symptoms, this supporting previous results obtained with Xff strain M23 [14] treated with Dentamet. It is worth noting that Dentamet^®^ showed a remarkable bactericidal activity also towards *Pantoea carbekii*, the primary bacterial symbiont of the brown marmorated stink bug *Halyomorpha halis* [22].

The antibacterial activity of metal ions against bacteria is known. In the model bacterium *Escherichia coli*, copper inactivates isopropylmalate dehydratase, an enzyme of the iron-sulphur dehydratase family [23], and, additionally, increases the amount of reactive oxygen species (ROS), this leading to DNA damage and inhibition of enzyme activities [24], whereas zinc incites a protein disfunction and loss of enzyme activity as well [24].

It should be noted that in the in vitro assay for each Dentamet^®^ dilution, the concentration of gDNA of all Xf strains was lower than those inoculated and detected at T0 (−ΔCt negative values), possibly suggesting that the bioproduct leads to a direct gDNA damage. Previously, an increase of mutations ROS-mediated, was observed for copper in *E. coli* [24]. Moreover, the bacteriolytic activity of copper and zinc towards bacteria is also known. Copper inhibits the biosynthesis of elongation of the peptidoglycan cell wall, whereas zinc acts on the activation of peptidoglycan autolysins [25] influencing the bacteria cell physiology and ability to growth. Previous studies showed that both Cu and Zn influence the *X. fastidiosa* state as biofilm and planktonic growth, and at concentrations of copper and zinc higher than 200 μM and 0.25 mM, respectively, the *X. fastidiosa* biofilm formation resulted inhibited [26,27]. In our condition, the biocomplex at 1/100 dilution corresponds at Zn 2.5 mM whereas an inhibitory effect is obtained at a lower Zn concentration (400 µM) in Navarrete and De La Fuente [28]. This could be explained by the fact that Navarrete and De La Fuente used ZnSO4 [28], whereas Dentamet^®^ exerts its activity through a ionic form of Zn. Most probably, ZnSO4 acts more effectively towards *X. fastidiosa* than Zn ions. In addition, for the full activation of *X. fastidiosa* virulence, the detoxification of zinc is required [28], suggesting that an additional supplement of the ion could act as a hindrance for the xylem pathogen colonization. Moreover, zinc, at 0.01–0.1 mM, inhibited the biofilm production in *Streptococcus mutans* [29]. With regards to these studies, we tested, in a liquid bacterial culture, the efficacy of the biocomplex in inhibiting the *X. fastidiosa* biofilm formation. Our data showed that the compound significantly inhibited the biofilm production of Xfp (De Donno, CFBP 8402), Xff (Temecula1) and Xfm (CFBP 8416) strains in a dose-dependent manner, and up to a dilution of 1:100 respect to the untreated control.

Previously, the in vitro efficacy of fosetyl-aluminium nanocrystals towards representative strains of the three subspecies (Xfp, Xff, Xfm) has been reported [30]; it is worth noting that, according to the same assays, Dentamet^®^ seems to be more effective in both the antibacterial activity and biofilm inhibition. Recently, Ge et al. [31] showed that the amendment of copper to potted tobacco plants did not prevent *X. fastidiosa* subsp. *fastidiosa* infection and hypothesized that the plants could have restricted copper uptake through copper homeostatic mechanisms so that the bacterium could have taken advantage and started its growth.

In agreement with our results in vitro and in the case olive trees treated with Dentamet^®^, it cannot be excluded also a synergistic effect played by the two ions in inhibiting the growth of the tested Xfp, Xff and Xfm strains, as reported for other bacterial species for which the toxic effect of the mixture was higher than those of the individual ions [32,33,34,35]. Dentamet^®^ is a mixture of zinc, copper and citric acid. Therefore, a synergistic effect could be more effective than the effect of the single ions, as studied by Ge et al. [31]. It is worth noting that a synergistic inhibition effect of low, non-toxic concentration of copper and organic acids is reported towards bacterial pathogens of humans (*Salmonella enterica, Pseudomonas aeruginosa* and *Vibrio cholerae*) and of plants (*Erwinia amylovora, Xanthomonas euvesicatoria, Pseudomonas syringae*) [36]. This effect on bacterial growth is due to the ability of organic acid to shuttle ions through the bacterial membrane, leading to an increasing of the ion effect on bacterial cells [25,36].

The in vitro efficacy of the biocomplex in inhibiting either the bacterial growth and the biofilm production would suggest its ability to act either in reducing the bacterial vessel extensive colonization (planktonic phase) and the biofilming phase that in bacterial pathogenesis, “is an often-critical behaviour linked to virulence and chronic bacterial infections” [37,38].

It can be suggested that Dentamet^®^, in which zinc, copper and organic acid are complexed, can exert its capacity to reduce the Xfp cell density in both cellular phenotypes (i.e., adhesive versus non-adhesive), also in vitro such as *in planta*, after repeated treatments to the crown, by reaching the olive xylem vessels and releasing the ions and citric acid which could exert their antibacterial efficacy through a synergistic effect. Note that the soil and leaf ionome comparison of olive groves located in Salento (i.e., Lecce, Taranto and Brindisi provinces) and north of Bari (Barletta-Andria-Trani Province) areas revealed a different composition. In particular, a lower content of both zinc and copper was found in the olive groves of the “infected” area of Salento [39]. These features further suggest the efficacy of biocomplex in providing the tree with ions that positively influence the plant defence systems to face Xfp. Other studies clearly indicated that also the leaf ionome of grape, blueberry and pecan plants infected by Xf resulted markedly changed [40]. In addition, D’Attoma et al. [41] found in olive groves of Salento area infected by Xfp an higher level of manganese in the leaf ionome composition of cultivar Leccino when compared to Ogliarola salentina.

Previously, we have reported the efficacy of Dentamet^®^ in reducing the field symptoms caused by *X. fastidiosa* subsp. *pauca* to olive trees located in Salento area. To corroborate such an evidence, we assessed the CFU equivalents of the pathogen within some of the treated olive trees over two years, and we found a significant reduction of the bacterial inoculum within the leaves [14].

In the present study, we tested the effectiveness of the compound towards a larger number of trees (i.e., a total of 41 trees) located in two olive groves of the “infected” area of Salento that applied the control strategy during a period of 3 and 4 years of application. The investigation aimed at recording the efficacy of the control strategy during the mid-term period in an area heavily affected by the OQDS [18].

The visual assessment of twig die-back symptoms carried out during 2019 exhibited a trend that shows the highest symptom incidence in March, after a period of six months during which the trees did not receive the treatments, and the lowest symptoms incidence in October 2019, after having received the six treatments. A similar trend was observed with the CFU equivalents assessment. Note that the Xfp concentration found in October 2018 within the treated trees resulted similar to those subsequently found within the same trees in March 2019, with Leccino showing the lowest Xfp concentration in comparison with the other cultivars. The bacterial concentrations of July and October were reduced when compared to March, with a significative reduction for Cellina di Nardò in Galatone and did not statistically differ from each other. Previously, it had been noted that stopping treatments in July and August had led to an increase in bacterium concentrations in the following months [14]; moreover, in almond, the effect of summer high temperatures induces an increase of tree colonization by the bacterium [42]. From these observations, it was inferred the need for treatments throughout the summer. The results herein obtained in July and October seem to confirm this feature, since the persistence of the treatments has kept the bacterial cell density low between July and October. Moreover, it has been also observed that all the non-treated neighbouring control trees (that resulted positive for Xfp before the starting of the biocomplex spraying) died during 2018–2019 in both plots, not allowing us to establish neither their twig wilting visual screening nor their Xfp cell density (Figure 5 and Figure 8), this stressing the high incidence and severity of the disease in the area. This suggests that repeated cycle of Dentamet treatments enabled to maintain the bacterium concentration to the point that the trees survive to the recurrent pathogen infections.

In general, a higher inoculum pressure was observed in Galatone compared to Cannole as revealed by both the visual and the cell density assessments (Figure 4 and Figure 9). In particular, the cultivars Ogliarola salentina and Cellina di Nardò showed higher concentrations in Galatone (in a mean range of 1.5–4.5 × 10^4^ CFU equivalents g^−1^ per leaf) than in Cannole (3.6–3.7 × 10^3^ CFU equivalents g^−1^). This difference could be due to the additional one-year-round of treatments carried out at Cannole. It is worth noting that bacterial concentrations of the two sensitive cultivars found in Cannole orchard in March (3.6–3.7 × 10^3^ CFU equivalents g^−1^ per leaf for Ogliarola salentina and Cellina di Nardò, respectively) were comparable to the concentration found in the same period on the resistant Leccino cv. (2.2 × 10^3^ CFU equivalents g^−1^ per leaf) in Galatone. Moreover, it is relevant to note also that Cellina di Nardò, that in Galatone, showed a higher pathogen density in March (4.5 × 10^4^ CFU equivalents g^−1^ per leaf) and subsequently showed a decrease of Xfp concentrations (4 × 10^2^ CFU equivalents g^−1^ per leaf in July and 4.4 × 10^3^ CFU equivalents g^−1^ per leaf CFU g^−1^ in October). This suggests once more the effectiveness of the control strategy in the mid-term period in reducing the Xfp cell densities within olive leaves. This finding acquires significance regardless of comparison with an untreated control evaluated in October 2018 that showed a bacterial concentration in the order of 10^6^ CFU equivalents g^−1^ of leaf in all cultivars, in both plots. Moreover, taking into account, as a reference, the data of the scientific literature, an average of bacterial concentration in 58 plants of 38 “infected” locations of the province of Lecce was reported as 10^4^–10^6^ CFU g^−1^ of leaf tissue depending on the different cultivars tested [43]. Similar results were confirmed by Giampietruzzi et al. [10] that reported two orders of magnitude lower (E + 04 vs. E + 06) in cv. Leccino than in cv. Ogliarola salentina. Finally, Luvisi et al. [44] agreed to preliminary results of bacterial concentration observed in Leccino (1.3 × 10^4^ CFU mL^−1^ tissue extract) and in Ogliarola di Lecce (2.1 × 10^5^ CFU mL^−1^ tissue extract). Basically, the untreated plants reported a bacterial concentration in the order of 10^6^ CFU g^−1^ for the susceptible cultivars and about 10^4^ CFU g^−1^ for the resistant Leccino, suggesting that the treatment with Dentamet^®^ reduced the bacterial concentration of the susceptible cultivars Ogliarola and Cellina at concentrations that are similar (about 10^4^ CFU g^−1^) or below (about 10^3^ CFU g^−1^) to the Xf concentrations generally founded in the resistant Leccino and reduced the bacterial concentration in the resistant Leccino of two order of magnitude up to about 10^2^ CFU g^−1^.

It is worth noting that, as in the cases of Galatone and for Cannole, the surrounding olive groves have been completely abandoned by the farmers, this resulting in a relevant increase of weeds that host *P. spumarius*. It has been estimated that in April, in Apulia, the density of *P. spumarius* nymphs ranges between 10 and 40 individuals per m^2^ [45,46] and that adults can move up to 100 m per day [47]. Consequently, it has been assumed that *X. fastidiosa* can be moved by a single vector between different host plants located up to 1 km of distance [8] and that the vector may inoculate olive trees during spring, summer and autumn over an extended period of time [46,48]. These features strongly indicate that the possibility of reaching the plots hosting the investigation by the insect vectors moving from the abandoned olive groves was very high during the years. Notwithstanding, the trees that received the biocomplex have faced the continuous re-infections transmitted by the nearby vectors and even started to yield.

The mid-term assessment of two olive groves located in the “infected” area of Salento that explore the Xfp control strategy based on the spray treatment of Dentamet^®^ to the crown has allowed to observe a satisfactory situation both in terms of vegetation and yield with the indirect effect of reduce the bacterium inoculum for the vector. It should be said that also the removal of weeds from February to April has retained another important aspect of the strategy since it is critical to reduce the juvenile forms of the vector in the olive grove [46]. The elimination of weeds could be effectively obtained through the regular mowing or superficial soil tilling. Furthermore, the massive pruning performed every 4–5 years that causes a relevant physiological stress to the tree should be avoided. This practice should be replaced by an annual or biennial pruning round that allows a better air circulation and light interception in the crown as well as a better management of the harvest [49]. Finally, soil fertility should be kept at a good level to enhance the control strategy [50]. It should be also noted that the amount of copper released upon the six treatments in one year is of about 500 g/ha, much less than 4 kg/ha that represents the current limit for copper amount allowed for organic agriculture. These considerations are part of a general strategy for the management of the olive groves which could lead to a cohabitation with the pathogen while preserving the Apulian landscape in areas not yet severely compromised by the disease [51].

## 4. Materials and Methods

### 4.1. Bacterial Strains Used in This Study

Pure cultures of *X. fastidiosa* subsp. *pauca* (De Donno_CFBP8402) (Xfp), isolated in Apulia (Italy) from olive trees, *X. fastidiosa* subsp*. fastidiosa* (Temecula1) (Xff), isolated in Temecula (CA, USA) from grapevine and *X. fastidiosa* subsp*. multiplex* (CFBP 8416) (Xfm) isolated in Propriano (Corsica, France) from *Polygala myrtifolia* (Xfm) were grown in PD2 agar medium for 15–0 days at 28 °C depending on the subspecies.

### 4.2. In Vitro Antibacterial Assays

Pure cultures of *X. fastidiosa* subspecies grown in PD2 agar medium as above reported, were scraped off, resuspended in PD2 broth and grown up to about 10^8^ colony forming units (CFU) mL^−1^ concentration (OD600 = 0.8).

For a preliminary assessment of the minimum bactericidal concentration (MBC) of the compound, serial ten-fold dilutions (from 1:10 to 1:100.000) of Dentamet^®^ in PD2 broth (10 mL) were inoculated with 100 µL of Xfp bacterial suspension. Each dilution was tested in triplicate, performing Real-Time PCR on bacterial suspensions at time 0 and after 6, 15 and 30 days.

Three Dentamet^®^ dilutions (1:10, 1:50 and 1:100) were selected to be further tested. PD2 broth (6mL) was inoculated with 60 µL of Xfp strain De Donno, Xff strain Temecula1 and Xfm strain CFBP8416 bacteria suspensions in polypropylene tubes. For each Dentamet^®^ dilution, three replications were performed. Negative controls consisted of sterile PD2 broth and PD2 broth supplemented with each of the Dentamet^®^ dilution. Positive control consisted in tubes containing only PD2 broth inoculated with Xfp strain De Donno, Xff strain Temecula1 and Xfm strain CFBP8416. The bacterial cultures were incubated at 28 °C, 170 rpm. Bacterial growth was followed for 6, 15- and 30-days post inoculation (dpi) for Xfp and up to 15 dpi for Xff and Xfm. Each time point was assessed by real-time PCR on DNA from bacterial cultures.

In parallel, three aliquots of 100 µl of each bacterial culture were plated on PD2 agar plates and incubated at 28–30 °C for 15–30 days, depending on the bacterial strain. The growth of typical bacterial colonies was controlled by microscope inspection, and at 15–30 days, the inoculated plate surface was scraped and suspended in distilled water to being assessed by real-time PCR to confirm the absence of growth.

### 4.3. Genomic DNA Extraction and Real-Time PCR

Genomic DNA (gDNA) was extracted from 700 µL of each bacterial culture tube or agar plates using the Gentra Puregene Yeast/Bact. Kit (Qiagen, Venlo, The Netherlands) according to the manufacturer’s instructions. The real-time PCR was performed by processing aliquots (1 μL) of gDNA by Francis et al., (2006), SYBRGreen version, following EPPO PM7/24 (4) by using SYBR™ Green PCR Master Mix (Applied Biosystem, Foster City, CA, USA). Each sample was assessed in duplicate.

### 4.4. Agar Dilution and Biofilm Inhibition Assays

Agar dilution assay was performed as reported by Bleve et al. [52] with slight modifications. In particular, PD2 agar medium was supplemented with Dentamet^®^ at final dilution of 1:10, 1:50 and 1:100 in polypropylene tubes. Each strain of Xfp, Xff and Xfm were grown in PD2 broth for 7 days at 28 °C and 100 rpm. A volume of 10 µL of decimal dilutions of bacterial suspensions from 10^7^ to 10^3^ CFU mL^−1^ of each inoculum were plated to the PD2 agar plates supplemented or not with Dentamet^®^. The agar plates were incubated at 28 °C for 8 days for Xff and Xfm and 20 days for Xfp. Four replications were performed for each inoculum concentration, and each experiment was carried out two times (*n* = 8). Images were captured by using a stereomicroscope (Wild Heerbrugg, Switzerland) connected to a digital camera (Leica IC80 HD) in the same conditions of bright field illumination. To further investigate the antibacterial activity of Dentamet^®^, after 30 days of growth for Xfp and 15 days for Xff and Xfm, the biofilm production was evaluated, through the crystal violet assay according to the method of Zaini et al. [53], with little modifications. In particular, the PD2 broth was removed, and the tubes were gently rinsed twice with sterile distilled water. Biofilm was stained with 500 µL of 0.01% crystal violet by manually washing the ring until a visual staining and, subsequently, treating it with 1 mL of absolute ethanol. The absorbance at 600 nm of the resulting ethanol solution was measured with DeNovix Spectrophotometer DS-11 Fx+ (Denovix Inc., Wilmington, DE, USA).

### 4.5. Statistical Analysis

The sample mean ± the standard deviation (SD) was calculated for all data. A one-way ANOVA Tukey’s test was applied for pairwise comparisons between means of different groups; the null hypothesis that the means are equal was assessed for each couple of normally distributed populations. Statistically significant difference between two subsets of data gives a significance level P (*p* value) less than 0.05. One-sample *T*-Test and Wilcoxon was applied for the statistical analysis of data reported in Figure 7. Analyses were performed trough GraphPad Prism.

Significant differences about bacterial DNA concentration and wilted twigs level were assessed, after verifying hypothesis of normality and equal variance, through Multiple ANOVA (*p*-value < 0.05) followed by post-hoc Tukey’s test (alpha = 0.05). Analyses were performed through R software.

### 4.6. Choice of the Olive Trees

The olive groves were chosen in order to assess the effectiveness of the control strategy in the mid-term period, upon three and four consecutive years of Dentamet^®^ applications. For this purpose were chosen two olive plots, with relatively young and old trees, that are part of typical olive groves of Salento (i.e., free vase training system, not regular pruning, ample space between the trees, no irrigation and soil fertilization, control of main pest and pathogens).

They have been also selected because, due to the Xfp outbreaks in the area, they are located in sites where the olive trees, including the bordering ones, showed clear symptoms of OQDS (i.e., twig and branch die-backs) at the beginning of the treatments.

Both plots applied to the tree canopy the biocomplex, once per month through nebulisation by using an atomizer from April to September, at a dose of 0.5% (*v:v*) [14]. One plot was also selected because, in addition to the widespread and Xfp-susceptible local cultivars Ogliarola salentina and Cellina di Nardò, hosts also Leccino trees, a cultivar that has been retained resistant to the Xfp population currently present in Salento [10]. Both plots were pruned (i.e., removal of all wilted parts and re-establishment of the free vase training system) during winter preceding the starting of the control strategy. Consequently, to allow a natural resprouting of the tree canopy, during the first years (i.e., 2 years for Galatone and 3 years for Cannole) the samplings were not performed. In each tree of both plots and nearby trees, the occurrence of Xfp was checked before the first spraying of the compound by following the procedures described elsewhere [14].

Plot A is located in Galatone (Lecce province; Lat.: 40.162242; Long.: 18.073356). It is planted with Ogliarola salentina, Cellina di Nardò and Leccino cultivars, of about 70 years-old, spaced at 6 × 6 m. The control strategy started on March 2017. Plot B is located in Cannole (Lecce province; Lat.: 40.152354; Long.: 18.404276). It is planted with 22 years old Ogliarola salentina and Cellina di Nardò cultivars, spaced at 8 × 8 m. The control strategy started on March 2016.

In March, July and October 2019, trees of both plots were assessed for scoring the new wilted twigs according to Scortichini et al. [14]. The assessment of March 2019 was carried out before the first spraying of the biocomplex. In addition, for each single tree of both plots, the olives were weighed in autumn 2019 for checking the yield of the plots after the reestablishment of the tree productivity. Xfp cell densities and field symptoms assessment were obtained in parallel from the same trees (i.e., from 10 Ogliarola salentina and 10 Cellina di Nardò trees in Cannole and from 7 Ogliarola salentina, Cellina di Nardò and Leccino trees in Galatone). The cell density was also evaluated in October 2018 before the 2019 assessment as T0.

In both plots, before starting the Xfp cell concentration assessment, 30 trees of the neighbouring farms that did not apply the control strategy were chosen as non-treated control: 15 at Galatone (five trees for Leccino, Cellina di Nardò and Ogliarola salentina) and 15 at Cannole (seven trees of Cellina di Nardò and eight trees of Ogliarola salentina) and were assessed for ascertaining both the occurrence and the density of Xfp using real-time PCR in October 2018. However, in March 2019, in both plots, the trees previously chosen as negative controls resulted severely damaged (i.e., most of the branches withered) (Figure 8), leading to a completely death of the plants from March to October 2019, and, consequently, it was not possible to assess their Xfp concentration within the leaves.

### 4.7. Quantitative Real-Time Assessment of Xfp within Olive Leaves

Sampling was performed by following the European and Mediterranean Plant Protection Organization recommendations EPPO Standard PM 7/24 (4) [18].

In particular, two independent samples were collected from basal (B) and medial (M) portion of each treated plant, for a total of eight sampling per plant; each sample was constituted of pooled twigs, with mature leaves, taken randomly from 4 cardinal points.

Each laboratory-sample consisted in about 25 mature leaves. gDNA was extracted with DNeasy mericon Food Kit (Qiagen, Venlo, The Netherlands) from 0.8 g of leaves central ribs randomly taken per laboratory-sample. gDNA was eluted in 70 μL elution buffer. The real-time PCR was performed according of Harper et al. (2010) [54]. Real-time PCR for each sample was performed in triplicate.

For Xfp DNA quantification purposes, a standard quantification procedure was performed based on the real-time PCR of Harper et al. (2010) [54] in each run by comparing the standard curves of gDNA of Xfp De Donno strain (from 10 ng to 10 fg) and a dilution series of olive extracts spiked with serial dilutions of bacterial suspension (10^7^–10 CFU mL^−1^) performed in triplicate. Based on the relationship between the amount of Xfp DNA detected and the concentration of added CFU, quantification of the pathogen was expressed in CFU equivalents [55].

Given that the weight of leaf samples was determined, and fixed volumes were used for DNA elution and for real-time PCR, CFU was referred as CFU g^−1^ of leaf sample.

Each sample is represented as basal (B) and medial (M) CFU g^−1^ ratio. Supplemental Tables S1–S3 show Ct values and *X. fastidiosa* DNA quantification correlated to CFU equivalents g^−1^.

Due to the death of the non-treated neighbouring control plants during 2019, in this assay, it was not possible to include viable non-treated samples, representative for each plant. As reported in the EPPO Standard PM 7/24 (4), necrotic and dead tissue must be removed from symptomatic leaves, and consequently, representative non-treated samples were not available for the comparison of the *X. fastidiosa* cell density data. As reference, the bacterial concentration assessed in 2016 in both plot and in 2018 in neighbouring plants located Cannole and in Galatone was taken in consideration together with evidence of other studies performed in the “infected” areas of the Lecce province and obtained with Ogliarola salentina, Cellina di Nardò and Leccino trees naturally infected by Xfp [10,41,43].

## Figures and Tables

**Table 1:** Mean, standard deviation (SD), interquartile range (IQR) and median of bacterial concentration of all collected samples, expressed as CFU equivalents g^−1^ of leaf. The data are reported considering each sampling (cultivars, months, and plots are shown). The data are also recorded by considering the concentrations of all samples for months, for plots and for the three different cultivars collected in Galatone. The high standard deviation depends on the differences between the bacterial concentration of the samples collected even within the same plant, related to the heterogeneous distribution of *X. fastidiosa* in the host plant (https://efsa.onlinelibrary.wiley.com/doi/pdf/10.2903/j.efsa.2015.3989; https://www.cabi.org/isc/datasheet/5719) (see Table S1 for more details).

CFU g^ (−1) 7 leaf						
Cultivar	Month ^a^	Plot	Mean	SD ^b^	Median	IQR ^c^
Ogliarola ^d^	March	Galatone	1.5 × 10^4^	1.5 × 10^4^	1.2 × 10^4^	1.6 × 10^4^
Ogliarola	July	Galatone	8.3 × 10^3^	1.6 × 10^3^	2.7 × 10^3^	5.3 × 10^3^
Ogliarola	October	Galatone	3.1 × 10^3^	3.7 × 10^3^	1.4 × 10^3^	4.6 × 10^3^
Ogliarola	March	Cannole	3.7 × 10^3^	5.9 × 10^3^	2.6 × 10^3^	4.0 × 10^3^
Ogliarola	July	Cannole	4.4 × 10^2^	5.9 × 10^2^	1.4 × 10^2^	7.2 × 10^2^
Ogliarola	October	Cannole	1.9 × 10^3^	4.4 × 10^3^	3.9 × 10^2^	5.0 × 10^3^
Cellina ^e^	March	Galatone	4.5 × 10^4^	4.4 × 10^4^	2.6 × 10^4^	4.0 × 10^4^
Cellina	July	Galatone	4.4 ×10^2^	5.9 × 10^2^	1.4 × 10^2^	7.2 × 10^2^
Cellina	October	Galatone	4.4 × 10^3^	3.3 × 10^3^	3.9 × 10^3^	5.0 × 10^3^
Cellina	March	Cannole	3.6 × 10^3^	6.2 × 10^3^	1.9 × 10^0^	4.9 × 10^3^
Cellina	July	Cannole	1.2 × 10^2^	3.5 × 10^2^	9.2 × 10^0^	1.2 × 10^2^
Cellina	October	Cannole	1.7 × 10^3^	2.6 × 10^3^	7.5 × 10^2^	1.8 × 10^2^
Leccino	March	Cannole	2.2 × 10^3^	3.1 × 10^3^	7.5 × 10^2^	3.7 × 10^3^
Leccino	July	Cannole	1.8 × 10^2^	3.5 × 10^2^	9.2 × 10^0^	1.3 × 10^2^
Leccino	October	Cannole	3.5 × 10^2^	7.9 × 10^2^	0	1.8 × 10^2^
	March ^f^		1.2 × 10^4^	2.5 × 10^4^	4.5 × 10^3^	1.4 × 10^4^
	July ^f^		2.1 × 10^3^	7.4 × 10^3^	1.2 × 10^2^	1.1 × 10^2^
	October ^f^		2.2 × 10^3^	3.4 × 10^3^	7.8 × 10^2^	2.4 × 10^3^
		Galatone ^g^	9.0 × 10^3^	2.1 × 10^4^	1.4 × 10^3^	7.5 × 10^3^
		Cannole ^g^	1.9 × 10^3^	4.2 × 10^3^	2.4 × 10^2^	1.5 × 10^3^
Ogliarola in Galatone ^h^			8.7 × 10^3^	1.4 × 10^4^	4.0 × 10^3^	1.0 × 10^4^
Cellina in Galatone ^h^			1.7 × 10^4^	3.2 × 10^4^	4.6 × 10^3^	1.6 × 10^4^
Leccino in Galatone ^h^			9.1 × 10^2^	2.1 × 10^3^	4.4 × 10^0^	5.9 × 10^2^

^a^ Month of sample collection. ^b^ Standard deviation. ^c^ Interquartile range. ^d^ Ogliarola salentina. ^e^ Cellina di Nardò. ^f^ Total mean calculated for months. ^g^ Total mean calculated for each plot. ^h^ Total mean calculated for each cultivar in Galatone.

## Supplementary Materials

The following are available online at https://www.mdpi.com/2076-0817/10/1/85/s1, Figure S1: *X. fastidiosa* subsp. *pauca* strain De Donno (CFBP8402), grown in addition with 1:10, 1:50 and 1:100 Dentamet^®^ dilutions and without Dentamet^®^ (control); Figure S2: Xfp Biofilm state growth. Table S1: The table reports the Ct value, X. fastidiosa gDNA ng μL-1 concentration, total X. fastidiosa gDNA amount, total X. fastidiosa gDNA amount per g sample, X. fastidiosa CFU equivalents per g sample and X. fastidiosa CFU equivalents per g plant sample. Each real time PCR run reports own standard curve parameters of efficiency (E), r2 and slope. The data are reported considering March samplings (cultivars and plots are shown); Table S2: The table reports the Ct value, X. fastidiosa gDNA ng μL-1 concentration, total X. fastidiosa gDNA amount, total X. fastidiosa gDNA amount per g sample, X. fastidiosa CFU equivalents per g sample and X. fastidiosa CFU equivalents per g plant sample. Each real time PCR run reports own standard curve parameters of efficiency (E), r2 and slope. The data are reported considering July samplings (cultivars and plots are shown); Table S3: The table reports the Ct value, X. fastidiosa gDNA ng μL-1 concentration, total X. fastidiosa gDNA amount, total X. fastidiosa gDNA amount per g sample, X. fastidiosa CFU equivalents per g sample and X. fastidiosa CFU equivalents per g plant sample. Each real time PCR run reports own standard curve parameters of efficiency (E), r2 and slope. The data are reported considering October samplings (cultivars and plots are shown).

## References

[1] Saponari M., Boscia D. (2013). Identification of DNA sequences related to *Xylella fastidiosa* in oleander, almond and olive trees exhibiting leaf scorch symptoms in Apulia (southern Italy). J. Plant. Pathol..

[2] Cariddi C., Saponari M. (2014). Isolation of Xylella fastidiosa strain infecting olive and oleander in Apulia, Italy. Plant Pathol. J..

[3] Martelli G.P. (2015). The current status of the quick decline syndrome of olive in southern Italy. Phytoparasitica.

[4] Scortichini M., Cesari G. (2019). An Evaluation of Monitoring Surveys of the Quarantine Bacterium *Xylella Fastidiosa* Performed in Containment and Buffer Areas of Apulia, Southern Italy. Appl. Biosaf..

[5] Scholten R., Martinez Sanchez L. Monitoring the impact of *Xylella* on Apulia’s olive orchards using MODIS satellite data supported by weather data. 2019. 2nd European Conference on *Xylella fastidiosa*, Ajaccio 29–30 October. http://www.efsa.europa.eu/sites/default/files/event/191029-xylella/S6.P1_BECK.pdf.

[6] Saponari M., Loconsole G., Cornara D., Yokomi R.K., De Stradis A., Boscia D., Bosco D., Gp G.P.M., Krugner R., Porcelli F. (2014). Infectivity and Transmission of *Xylella fastidiosa* by *Philaenus spumarius* (Hemiptera: Aphrophoridae) in Apulia, Italy. J. Econ. Èntomol..

[7] Fierro A., Liccardo A., Porcelli F. (2019). A lattice model to manage the vector and the infection of the *Xylella fastidiosa* on olive trees. Sci. Rep..

[8] Strona G., Carstens C.J., Beck P.S.A. (2017). Network analysis reveals why *Xylella fastidiosa* will persist in Europe. Sci. Rep..

[9] Schneider K., Van der Werf W. (2020). Impact of *Xylella fastidiosa* subspecies pauca in European olives. Proc. Natl. Acad. Sci. USA.

[10] Giampetruzzi A., Morelli M., Saponari M., Loconsole G., Chiumenti M., Boscia D., Savino V.N., Martelli G.P., Saldarelli P. (2016). Transcriptome profiling of two olive cultivars in response to infection by the CoDiRO strain of *Xylella fastidiosa* subsp. pauca. BMC Genom..

[11] Boscia D., Altamura G. (2017). Resistenza a *Xylella fastidiosa* in diverse cultivar di olivo. L’Informatore Agrario.

[12] Terra e vita. https://terraevita.edagricole.it/featured/xylella-mango-avocado/.

[13] Catalano L., Al-Dobai S. (2019). Guidelines for the Prevention, Eradication and Containment of Xylella Fastidiosa in Olive-Growing Areas.

[14] Scortichini M., Chen J. (2018). A zinc, copper and citric acid biocomplex shows promise for control of *Xylella fastidiosa* subsp. pauca in olive trees in Apulia region (southern Italy). Phytopathol. Mediterr..

[15] Del Coco L., De Pascali S.A., Fanizzi F.P. (2014). NMR-Metabolomic Study on Monocultivar and Blend Salento EVOOs including Some from Secular Olive Trees. Food Nutr. Sci..

[16] Negro C., Aprile A., Luvisi A., Nicolì F., Nutricati E., Vergine M., Miceli A., Blando F., Sabella E., De Bellis L. (2019). Phenolic Profile and Antioxidant Activity of Italian Monovarietal Extra Virgin Olive Oils. Antioxidants.

[17] Colella C., Carradone R. (2019). Problem Setting and Problem Solving in the Case of Olive Quick Decline Syndrome in Apulia, Italy: A Sociological Approach. Phytophatology.

[18] (2019). PM 7/24 (4) *Xylella fastidiosa*. Eppo Bull..

[19] D’Attoma G., Morelli M., De La Fuente L., Cobine P.A., Saponari M., De Souza A.A., De Stradis A., Saldarelli P. (2020). Phenotypic Characterization and Transformation Attempts Reveal Peculiar Traits of *Xylella fastidiosa* Subspecies *pauca* Strain De Donno. Microorganisms.

[20] Marcelletti S., Scortichini M. (2016). Genome-wide comparison and taxonomic relatedness of multiple *Xylella fastidiosa* strains reveal the occurrence of three subspecies and a new Xylella species. Arch. Microbiol..

[21] Denancé N., Briand M., Gaborieau R., Gaillard S., Jacques M.A. (2019). Identification of genetic relationships and subspecies signatures in *Xylella fastidiosa*. BMC Genom..

[22] Gonella E., Orrù B., Alma A. (2019). Egg masses treatment with micronutrient fertilizers has a suppressive effect on newly-emerged nymphs of the brown marmorated stink bug *Halyomorpha halys*. Èntomol. Gen..

[23] Macomber L., Imlay J.A. (2009). The Iron-Sulfur clusters of dehydratases are primary intracellular targets of copper toxicity. Proc. Natl. Acad. Sci. USA.

[24] Lemire J.A., Harrison J.J., Turner R.J. (2013). Antimicrobial activity of metals: Mechanisms, molecular targets and applications. Nat. Rev. Genet..

[25] Ishida T. (2017). Antibacterial Mechanism of Bacteriolyses of Bacterial Cell Walls by Zinc(2+) Ion Induced Activations of PGN Autolysins, and DNA damages. J. Genes Proteins..

[26] Cobine P.A., Cruz L.F., Navarrete F., Duncan D., Tygart M., De La Fuente L. (2013). *Xylella fastidiosa* Differentially Accumulates Mineral Elements in Biofilm and Planktonic Cells. PLoS ONE.

[27] Navarrete F., De La Fuente L. (2014). Response of *Xylella fastidiosa* to Zinc: Decreased Culturability, Increased Exopolysaccharide Production, and Formation of Resilient Biofilms under Flow Conditions. Appl. Env. Microbiol..

[28] Navarrete F., De La Fuente L. (2015). Zinc Detoxification Is Required for Full Virulence and Modification of the Host Leaf Ionome by *Xylella fastidiosa*. Mol. Plant Microbe Interact..

[29] Phan T.-N., Buckner T., Sheng J., Baldeck J.D., Marquis R.E. (2004). Physiologic actions of zinc related to inhibition of acid and alkali production by oral streptococci in suspensions and biofilms. Oral Microbiol. Immunol..

[30] Baldassarre F., Tatulli G., Vergaro V., Mariano S., Scala V., Nobile C., Pucci N., Dini L., Loreti S., Ciccarella G. (2020). Sonication-Assisted Production of Fosetyl-Al Nanocrystals: Investigation of Human Toxicity and In Vitro Antibacterial Efficacy against *Xylella fastidiosa*. Nanomaterials.

[31] Ge Q., Cobine P.A., De La Fuente L. (2020). Copper Supplementation in Watering Solution Reaches the Xylem But Does Not Protect Tobacco Plants Against *Xylella fastidiosa* Infection. Plant Dis..

[32] Shuttleworth K.L., Unz R.F. (1991). Influence of metals and metal speciation on the growth of filamentous bacteria. Water Res..

[33] Cabrero A., Fernandez S., Mirada F., Garcia J., García J. (1998). Effects of copper and zinc on the activated sludge bacteria growth kinetics. Water Res..

[34] Utgikar V., Chaudhary N., Koeniger A., Tabak H.H., Haines J.R., Govind R. (2004). Toxicity of metals and metal mixtures: Analysis of concentration and time dependence for zinc and copper. Water Res..

[35] Şengör S.S., Gikas P., Moberly J.G., Peyton B.M., Ginn T.R. (2011). Comparison of single and joint effects of Zn and Cu in continuous flow and batch reactors. J. Chem. Technol. Biotechnol..

[36] Zhitnisky D., Rose J. (2017). The higly synergistic, broad spectrum, antibacterial activity of organic acids and transition metals. Sci. Rep..

[37] Roper C., Castro C. (2019). *Xylella fastidiosa*: Bacterial parasitism with hallmarks of commensalism. Curr. Opin. Plant Biol..

[38] Cardinale M., Luvisi A., Meyer J.B., Sabella E., De Bellis L., Cruz A.C., Ampatzidis Y., Cherubini P. (2018). Specific Fluorescence in Situ Hybridization (FISH) Test to Highlight Colonization of Xylem Vessels by *Xylella fastidiosa* in Naturally Infected Olive Trees (Olea europaea L.). Front. Plant Sci..

[39] Del Coco L., Migoni D., Girelli C.R., Angilè F., Scortichini M., Fanizzi F.P. (2020). Soil and Leaf Ionome Heterogeneity in *Xylella fastidiosa* Subsp. Pauca-Infected, Non-Infected and Treated Olive Groves in Apulia, Italy. Plants.

[40] De La Fuente L., Parker J.K. (2013). The Bacterial Pathogen *Xylella fastidiosa* Affects the Leaf Ionome of Plant Hosts during Infection. PLoS ONE.

[41] D’Attoma G., Morelli M., Saldarelli P., Saponari M., Giampetruzzi A., Boscia D., Savino V.N., De La Fuente L., Cobine P.A. (2019). Ionomic Differences between Susceptible and Resistant Olive Cultivars Infected by *Xylella fastidiosa* in the Outbreak Area of Salento, Italy. Pathogens.

[42] Cao T., Connell J.H. (2011). Influence of inoculation date on the colonisation of *Xylella fastidiosa* and the persistence of almond leaf scorch disease among almond cultivars. Plant Dis..

[43] Saponari M., Boscia D., Altamura G., Loconsole G., Zicca S., D’Attoma G., Morelli M., Palmisano F., Tavano D., Savino V.N. (2017). Isolation and pathogenicity of *Xylella fastidiosa* associated to the olive quick decline syndrome in southern Italy. Sci. Rep..

[44] Luvisi A., Aprile A. (2017). *Xylella fastidiosa* subsp. *pauca* (CoDiRO strain) infection in four olive (Olea europaea L.) cultivars: Profile of phenolic compounds in leaves and progression of leaf scorch symptoms. Phytopathol. Mediterr..

[45] Dongiovanni C., Cavalieri V., Bodino N., Tauro D., Di Carolo M., Fumarola G., Altamura G., Lasorella C., Bosco D. (2018). Plant Selection and Population Trend of Spittlebug Immatures (Hemiptera: Aphrophoridae) in Olive Groves of the Apulia Region of Italy. J. Econ. Èntomol..

[46] Bodino N., Cavalieri V., Dongiovanni C., Plazio E., Saladini M.A., Volani S., Simonetto A., Fumarola G., Di Carolo M., Porcelli F. (2019). Phenology, seasonal abundance and stage-structure of spittlebug (Hemiptera: Aphrophoridae) populations in olive groves in Italy. Sci. Rep..

[47] Weaver C.R., King D.R. (1954). Meadow Spittlebug.

[48] Cornara D., Saponari M., Zeilinger A.R., De Stradis A., Boscia D., Loconsole G., Bosco D., Martelli G.P., Almeida R.P.P., Porcelli F. (2017). Spittlebugs as vectors of *Xylella fastidiosa* in olive orchards in Italy. J. Pest Sci..

[49] Gucci R., Cantini C. (2000). Pruning and Training Systems for Modern Olive Growing.

[50] Berendson R.L., Pieterse C.M.J. (2012). The rhizosphere microbiome and plant health. Trends Plant Sci..

[51] Scortichini M. (2020). The multi-millenial olive agroecosystem of Salento (Apulia, Italy) threatened by *Xylella fastidiosa* subsp. *pauca*: A working possibility of restoration. Sustainability.

[52] Bleve G., Gallo A., Altomare C., Vurro M., Maiorano G., Cardinali A., D’Antuono I., Marchi G., Mita G. (2018). In vitro activity of antimicrobial compounds against *Xylella fastidiosa*, the causal agent of the olive quick decline syndrome in Apulia (Italy). FEMS Microbiol. Lett..

[53] Zaini P.A., De La Fuente L. (2009). Grapevine Xylem Sap Enhances Biofilm Development by *Xylella Fastidiosa*. FEMS Microbiol. Lett..

[54] Harper S.J., Ward L.I., Clover G.R.G. (2010). Development of LAMP and Real-Time PCR Methods for the Rapid Detection of *Xylella fastidiosa* for Quarantine and Field Applications. Phytopathology.

[55] Modesti V., Pucci N., Lucchesi S., Campus L., Loreti S. (2016). Experience of the Latium region (Central Italy) as a pest-free area for monitoring of *Xylella fastidiosa*: Distinctive features of molecular diagnostic methods. Eur. J. Plant Pathol..

