# Supplementary material for: Further In Vitro Assessment and Mid-Term Evaluation of Control Strategy of Xylella fastidiosa subsp. pauca in Olive Groves of Salento (Apulia, Italy)
Source: Pathogens. 2021 Jan 19;10(1):85. doi: 10.3390/pathogens10010085 (PMC7835972; doi:10.3390/pathogens10010085)
Supplement: Supplementary file 1 [file pathogens-10-00085-s001.pdf]

Supplementary data.

Figure S1. *X. fastidiosa* subsp. *pauca* strain De Donno (CFBP8402), grown in addition with 1:10, 1:50 and 1:100 Dentamet® dilutions and without Dentamet® (control). Planktonic growth was assessed by real-time PCR on bacterial suspension. -ΔCt represents the difference between the Ct value obtained at each time point (respectively 6, 15, 30 dpi) and the Ct value at time 0 for each sample. Values are means ± SD of the most representative experiment of two independent experiments (n=8 in total). No amplification (N.A.) was observed in 1:10 and 1:50 Dentamet®. A statistically significant difference was obtained between controls Xfp, (grown in PD2 broth) and the same strain added with Dentamet® 1:100 dilution, according to one-way ANOVA, Dunnett's test (****p≤ 0.0001 vs Ctr).

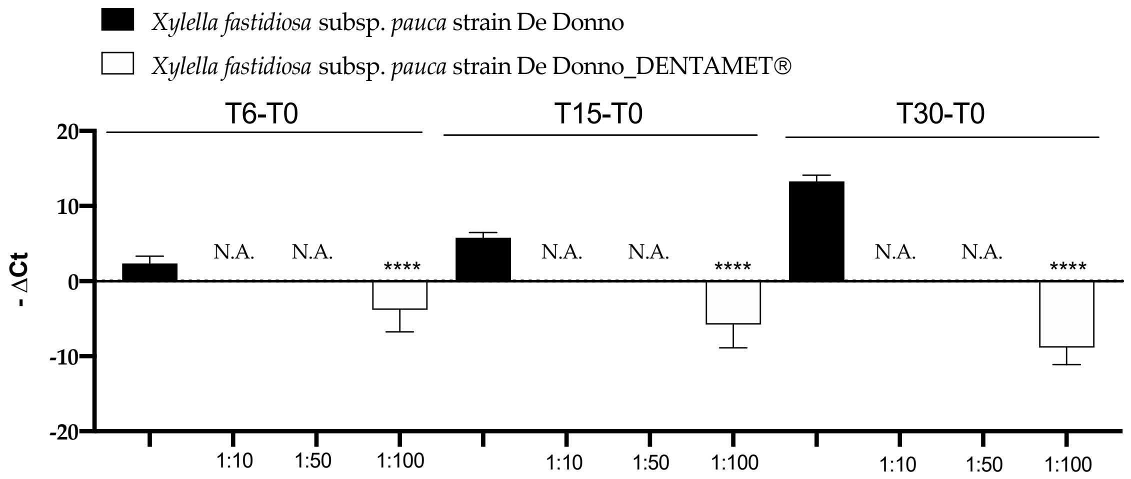

Figure S2. **Xfp Biofilm state growth**. X. *fastidiosa* biofilm state, in control tubes, is represented as a ring on tube walls (Crystal violet assay). No biofilm growth was observed for all the Dentamet® dilutions.
This figure is representative for all X. *fastidiosa* subspecies.

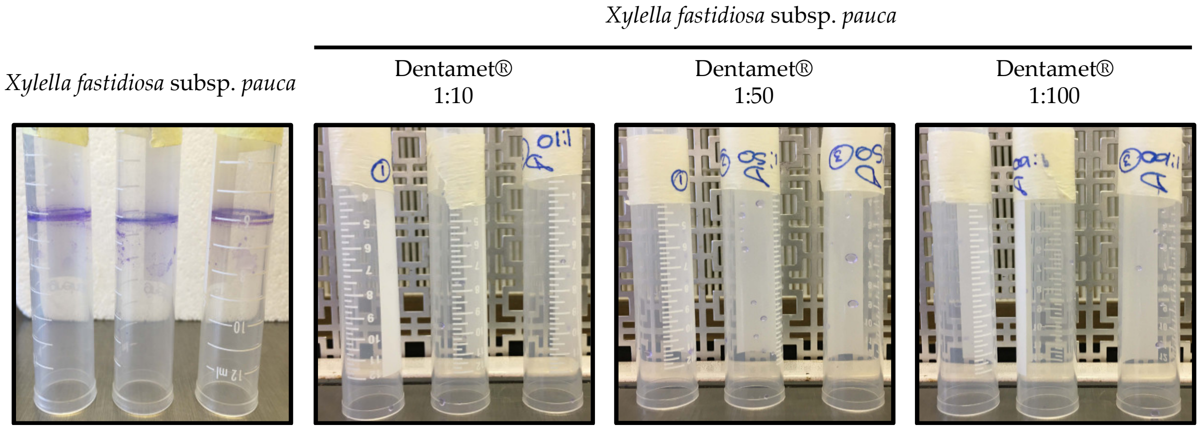

Table S1. The table reports the Ct value, X. *fastidiosa* gDNA ng μL^{-1} concentration, total X. *fastidiosa* gDNA amount, total X. *fastidiosa* gDNA amount per g sample, X. *fastidiosa* CFU equivalents per g sample and X. *fastidiosa* CFU equivalents per g plant sample. Each real time PCR run reports own standard curve parameters of efficiency (E), r^2 and slope. The data are reported considering March samplings (cultivars and plots are shown).

Cultivar	Plot	CT ratio	ng μL^{-1}	ng TOT	ng Tot g^{-1} sample	CFU g^{-1}	CFU value g^{-1} plant
OGLIAROLA 1 B	CANNOLE	28.83	0.01083	0.75831	0.94789	4.75E+03	2.38E+03
OGLIAROLA 1 M	CANNOLE	37.27	0.00004	0.00296	0.00371	1.86E+01	
OGLIAROLA 2 B	CANNOLE	32.35	0.00150	0.10475	0.13094	6.56E+02	5.68E+03
OGLIAROLA 2 M	CANNOLE	27.65	0.02441	1.70	2.13	1.07E+04	
OGLIAROLA 3 B	CANNOLE	36.30	0.00008	0.00560	0.00699	3.51E+01	4.92E+01
OGLIAROLA 3 M	CANNOLE	35.93	0.00014	0.01012	0.01265	6.34E+01	
OGLIAROLA 4 B	CANNOLE	33.19	0.00062	0.04357	0.0547	2.73E+02	1.63E+02
OGLIAROLA 4 M	CANNOLE	35.65	0.00012	0.00857	0.01071	5.37E+01	
OGLIAROLA 5 B	CANNOLE	35.39	0.00014	0.01013	0.01266	6.35E+01	1.92E+03
OGLIAROLA 5 M	CANNOLE	29.17	0.00861	0.60257	0.75321	3.77E+03	
OGLIAROLA 6 B	CANNOLE	36.48	0.00009	0.00611	0.00764	3.83E+01	6.24E+01
OGLIAROLA 6 M	CANNOLE	34.97	0.00020	0.01379	0.01724	8.64E+01	
OGLIAROLA 7 B	CANNOLE	37.46	0.00004	0.00262	0.00328	1.64E+01	8.22E+00
OGLIAROLA 7 M	CANNOLE	0.00	0.00	0.00	0.00	0.00E+00	
OGLIAROLA 8 B	CANNOLE	36.73	0.00006	0.00440	0.00550	2.76E+01	4.36E+03
OGLIAROLA 8 M	CANNOLE	27.97	0.01982	1.38	1.73	8.69E+03	
OGLIAROLA 9 B	CANNOLE	27.04	0.03473	2.43	3.03	1.52E+04	9.96E+03
OGLIAROLA 9 M	CANNOLE	28.84	0.01071	0.74939	0.93674	4.60E+03	
OGLIAROLA 10 B	CANNOLE	26.94	0.03699	2.58	3.23	1.62E+04	8.19E+03
OGLIAROLA 10 M	CANNOLE	33.97	0.00037	0.02621	0.03276	1.62E+02	
CELLINA 1 B	CANNOLE	28.64	0.01236	0.86494	1.08	5.42E+03	4.00E+03
CELLINA 1 M	CANNOLE	30.02	0.00587	0.41103	0.51379	2.58E+03	
CELLINA 2 B	CANNOLE	28.53	0.01332	0.93207	1.16	5.84E+03	1.04E+04
CELLINA 2 M	CANNOLE	27.07	0.03413	2.38	2.98	1.50E+04	
CELLINA 3 B	CANNOLE	0.00	0.00	0.00	0.00	0.00E+00	3.14E+03 *[1]
CELLINA 10 M	CANNOLE	26.56	0.04760	3.32	4.16	2.09E+04	1.30E+04 *[2]

E=92.6%; r^2= 0.980; Slope=-3.515

*[1] Mean with Cellina di Nardò 3M Cannole

*[2] Mean with Cellina di Nardò 10B Cannole

Cultivar	Plot	CT ratio	ng μL^{-1}	ng TOT	ng Tot g^{-1} sample	CFU g^{-1}	CFU value g^{-1} plant
CELLINA 3 M	CANNOLE	26.78	0.01430	1.00	1.25	6.27E+03	3.14E+03 *[1]
CELLINA 4 B	CANNOLE	28.80	0.00389	0.27207	0.34009	1.70E+03	3.34E+03
CELLINA 4 M	CANNOLE	27.14	0.01133	0.79307	0.99134	4.97E+03	
CELLINA 5 B	CANNOLE	36.18	0.00003	0.00217	0.00272	1.36E+01	1.20E+01
CELLINA 5 M	CANNOLE	36.68	0.00002	0.00166	0.00207	1.04E+01	
CELLINA 6 B	CANNOLE	36.09	0.00003	0.00230	0.00288	1.44E+01	1.88E+01
CELLINA 6 M	CANNOLE	35.37	0.00005	0.00369	0.00461	2.31E+01	
CELLINA7 B	CANNOLE	0.00	0.00	0.00	0.00	0.00E+00	0.00E+00
CELLINA 7 M	CANNOLE	0.00	0.00	0.00	0.00	0.00E+00	
CELLINA 8 B	CANNOLE	35.39	0.00005	0.00365	0.00456	2.29E+01	1.70E+01
CELLINA 8 M	CANNOLE	36.61	0.00003	0.00177	0.00221	1.11E+00	
CELLINA 9 B	CANNOLE	36.13	0.00004	0.00267	0.00333	1.67E+01	1.52E+01
CELLINA 9 M	CANNOLE	36.18	0.00003	0.00219	0.00273	1.37E+01	
CELLINA 10 B	CANNOLE	27.11	0.01155	0.80879	1.01	5.07E+03	1.30E+04 *[2]

E=92%; r^2= 0.996; Slope=-3.529

*[1] Mean with Cellina di Nardò 3B Cannole
*[2] Mean with Cellina di Nardò 10M Cannole

Cultivar	Plot	CT ratio	ng μL^{-1}	ng TOT	ng Tot g^{-1} sample	CFU g^{-1}	CFU value g^{-1} plant
LECCINO 1 B	GALATONE	31.42	0.00079	0.05510	0.06887	3.45E+02	3.22E+03
LECCINO 1 M	GALATONE	27.07	0.01391	0.97352	1.21	6.10E+03	
LECCINO 2 B	GALATONE	30.00	0.00200	0.14011	0.17513	8.78E+02	3.62E+03
LECCINO 2 M	GALATONE	27.01	0.01451	1.01	1.26	6.36E+03	
LECCINO 3 B	GALATONE	35.10	0.00008	0.00535	0.00668	3.35E+01	2.37E+03
LECCINO 3 M	GALATONE	27.47	0.01072	0.75040	0,75	4.70E+03	
LECCINO 4 B	GALATONE	30.52	0.00142	0.09916	0.12395	6.21E+02	3.27E+02
LECCINO 4 M	GALATONE	34.99	0.00007	0.00522	0.00653	3.27E+01	
LECCINO 5 B	GALATONE	34.34	0.00013	0.00907	0.01133	5.68E+01	3.25E+01
LECCINO 5 M	GALATONE	37.06	0.00002	0.00131	0.00164	8.23E+00	
LECCINO 6 B	GALATONE	26.39	0.02189	1.53	1.91	9.60E+03	5.35E+03
LECCINO 6 M	GALATONE	29.67	0.00250	0.17500	0.21875	1.10E+03	
LECCINO 7 B	GALATONE	35.29	0.00007	0.00490	0.00613	3.07E+01	5.28E+02
LECCINO7 M	GALATONE	29.77	0.00234	0.16380	0.20475	1.03E+01	
CELLINA 1 B	GALATONE	26.25	0.02398	16.68	20.98	1.05E+04	2.46E+04
CELLINA 1 M	GALATONE	24.28	0.08819	6.17	7.71	3.87E+04	
CELLINA 2 B	GALATONE	37.02	0.00002	0.00144	0.00180	9.03E+00	2.05E+04
CELLINA 2 M	GALATONE	24.19	0.09365	6.55	8.19	4.11E+04	
CELLINA 3 B	GALATONE	22.22	0.34644	24.25	30.31	1.52E+05	1.11E+05
CELLINA 3 M	GALATONE	23.38	0.16100	11.26	14.08	7.06E+04	

E=93.8%; r^2=0.996; Slope=-3.379

Cultivar	Plot	CT ratio	ng μL^{-1}	ng TOT	ng Tot g^{-1} sample	CFU g^{-1}	CFU value g^{-1} plant
CELLINA 4 B	GALATONE	26.5	0.02343	1.64	2.05	1.03E+04	3.92E+04
CELLINA 4 M	GALATONE	23.61	0.15530	10.87	13.58	6.81E+04	
CELLINA 5 B	GALATONE	25.34	0.05028	3.51	4.39	2.21E+04	7.18E+04
CELLINA 5 M	GALATONE	22.73	0.27696	19.38	24.23	1.21E+05	
CELLINA 6 B	GALATONE	25.22	0.05410	3.78	4.73	2.37E+04	2.21E+04
CELLINA 6 M	GALATONE	25.45	0.04649	3.25	4.06	2.04E+04	
CELLINA7 B	GALATONE	25.35	0.05101	3.57	4.46	2.24E+04	2.57E+04
CELLINA 7 M	GALATONE	24.91	0.06625	4.63	5.79	2.91E+04	
OGLIAROLA 1 B	GALATONE	25.76	0.03822	2.67	3.34	1.67E+04	1.13E+04
OGLIAROLA 1 M	GALATONE	27.34	0.01350	0.94487	1.18	5.92E+03	
OGLIAROLA 2 B	GALATONE	23.87	0.13088	9.16	11.45	5.74E+04	3.99E+04
OGLIAROLA 2 M	GALATONE	25.31	0.05116	3.58	4.47	2.24E+04	
OGLIAROLA 3 B	GALATONE	32.31	0.00056	0.03641	0.04551	2.28E+02	1.28E+02
OGLIAROLA 3 M	GALATONE	35.55	0.00006	0.00438	0.00545	2.73E+01	
OGLIAROLA 4 B	GALATONE	25.25	0.05311	3.71	4.64	2.33E+04	2.60E+04
OGLIAROLA 4 M	GALATONE	24.93	0.06530	4.57	5.71	2.86E+04	
OGLIAROLA 5 B	GALATONE	25.92	0.03416	2.39	2.98	1.50E+04	1.40E+04
OGLIAROLA 5 M	GALATONE	26.14	0.02959	2.07	2.58	1.30E+04	
OGLIAROLA 6 B	GALATONE	27.56	0.01167	0.81680	1.02	5.12E+03	2.94E+03
OGLIAROLA 6 M	GALATONE	30.45	0.00176	0.12301	0.15376	7.71E+02	
OGLIAROLA 7 B	GALATONE	26.97	0.01746	1.22	1.52	7.66E+03	9.52E+03
OGLIAROLA 7 M	GALATONE	26.34	0.02596	1.81	2.27	1.14E+04	

E=92.5%; r^2=0.992; Slope=-3.515

Table S2. The table reports the Ct value, X. *fastidiosa* gDNA ng μL^{-1} concentration, total X. *fastidiosa* gDNA amount, total X. *fastidiosa* gDNA amount per g sample, X. *fastidiosa* CFU equivalents per g sample and X. *fastidiosa* CFU equivalents per g plant sample. Each real time PCR run reports own standard curve parameters of efficiency (E), r^2 and slope. The data are reported considering July samplings (cultivars and plots are shown).

Cultivar	Plot	CT ratio	ng μL^{-1}	ng TOT	ng Tot g^{-1} sample	CFU g^{-1}	CFU value g^{-1} plant
CELLINA 1 B	CANNOLE	36.19	0.00002	0.00146	0.00182	9.12E+00	4.56E+00
CELLINA 1 M	CANNOLE	0.00	0.00	0.00	0.00	0.00E+00	
CELLINA 2 B	CANNOLE	36.08	0.00002	0.00156	0.00196	9.82E+00	4.91E+00
CELLINA 2 M	CANNOLE	0.00	0.00	0.00	0.00	0.00E+00	
CELLINA 3 B	CANNOLE	36.96	0.00001	0.00109	0.00127	6.38E+00	6.25E+02
CELLINA 3 M	CANNOLE	28.99	0.00283	0.19839	0.24799	1.24E+03	
CELLINA 4 B	CANNOLE	30.59	0.00095	0.06634	0.08293	4.16E+02	2.08E+02
CELLINA 4 M	CANNOLE	0.00	0.00	0.00	0.00	0.00E+00	
CELLINA 5 B	CANNOLE	0.00	0.00	0.00	0.00	0.00E+00	4.72E+02
CELLINA 5 M	CANNOLE	29.38	0.00215	0.15074	0.18843	9.44E+02	
CELLINA 6 B	CANNOLE	35.17	0.00004	0.00306	0.00383	1.91E+01	9.60E+00
CELLINA 6 M	CANNOLE	0.00	0.00	0.00	0.00	0.00E+00	
CELLINA7 B	CANNOLE	0.00	0.00	0.00	0.00	0.00E+00	1.61E+01
CELLINA 7 M	CANNOLE	34.47	0.00007	0.00512	0.00641	3.21E+01	
CELLINA 8 B	CANNOLE	0.00	0.00	0.00	0.00	0.00E+00	0.00E+00
CELLINA 8 M	CANNOLE	0.00	0.00	0.00	0.00	0.00E+00	
CELLINA 9 B	CANNOLE	36.19	0.000002	0.00145	0.00182	9.11E+00	1.02E+01
CELLINA 9 M	CANNOLE	35.87	0.00003	0.00180	0.00225	1.13E+01	
CELLINA 10 B	CANNOLE	30.55	0.00097	0.06772	0.08466	4.24E+02	3.79E+02
CELLINA 10 M	CANNOLE	30.26	0.00118	0.08240	0.10301	5.16E+02	
OGLIAROLA 1 B	CANNOLE	0.00	0.00	0.00	0.00	0.00E+00	2.71E+00
OGLIAROLA 1 M	CANNOLE	36.95	0.00001	0.00086	0.00108	5.42E+00	
OGLIAROLA 2 B	CANNOLE	32.71	0.00022	0.01555	0.01944	9.75E+01	1.37E+02
OGLIAROLA 2 M	CANNOLE	31.87	0.00040	0.02833	0.03541	1.78E+02	
OGLIAROLA 3 B	CANNOLE	0.00	0.00	0.00	0.00	0.00E+00	9.73E+02
OGLIAROLA 3 M	CANNOLE	28.32	0.00444	0.31061	0.38827	1.95E+03	
OGLIAROLA 4 B	CANNOLE	29.75	0.00167	0.11686	0.14608	7.32E+02	3.71E+02
OGLIAROLA 4 M	CANNOLE	36.02	0.00002	0.00162	0.00203	1.24E+01	
OGLIAROLA 5 B	CANNOLE	30.90	0.00077	0.05381	0.06726	3.37E+02	2.91E+02
OGLIAROLA 5 M	CANNOLE	31.39	0.00056	0.03899	0.04874	2.44E+02	
OGLIAROLA 6 B	CANNOLE	0.00	0.00	0.00	0.00	0.00E+00	3.44E+02
OGLIAROLA 6 M	CANNOLE	29.84	0.00157	0.10978	0.13722	6.88E+02	
OGLIAROLA 7 B	CANNOLE	36.01	0.00002	0.00164	0.00205	1.03E+01	2.28E+01 *[1]
OGLIAROLA 8 B	CANNOLE	29.77	0.00170	0.11888	0.14860	7.45E+02	1.03E+03 *[2]
OGLIAROLA 9 B	CANNOLE	28.81	0.00322	0.2256	0.28201	1.41E+03	1.44E+03 *[3]
OGLIAROLA 10 B	CANNOLE	0.00	0.00	0.00	0.00	0.00E+00	5.61E+00 *[4]

E=97.7%; r^2=0.986; Slope=-3.379

*[1] Mean with Ogliarola salentina 7M Cannole

*[2] Mean with Ogliarola salentina 8M Cannole

*[3] Mean with Ogliarola salentina 9M Cannole

*[4] Mean with Ogliarola salentina 10M Cannole

Cultivar	Plot	CT ratio	ng μL^{-1}	ng TOT	ng Tot g^{-1} sample	CFU g^{-1}	CFU value g^{-1} plant
OGLIAROLA 7 M	CANNOLE	34.38	0.00008	0.00565	0.00706	3.54E+01	2.28E+01 *[1]
OGLIAROLA 8 M	CANNOLE	28.91	0.00299	0.20932	0.26165	1.31E+03	1.03E+03 *[2]
OGLIAROLA 9 M	CANNOLE	28.74	0.00336	0.23485	0.29356	1.47E+03	1.44E+03 *[3]
OGLIAROLA 10 M	CANNOLE	36.10	0.00003	0.00179	0.00223	1.12E+01	5.61E+00 *[4]
CELLINA 1 B	GALATONE	30.58	0.00099	0.06924	0.08655	4.34E+02	6.38E+02
CELLINA 1 M	GALATONE	29.59	0.00192	0.13427	0.16784	8.41E+02	
CELLINA 2 B	GALATONE	32.65	0.00027	0.01855	0.02319	1.16E+02	3.57E+02
CELLINA 2 M	GALATONE	30.10	0.00136	0.9541	0.11927	5.98E+02	
CELLINA 3 B	GALATONE	27.02	0.01047	0.73308	0.91635	4.59E+03	2.71E+03
CELLINA 3 M	GALATONE	29.61	0.00188	0.13135	0.16419	8.23E+02	
CELLINA 4 B	GALATONE	26.08	0.01952	1.36	1.70	8.56E+03	4.84E+03
CELLINA 4 M	GALATONE	29.15	0.00256	0.17948	0.22435	1.12E+03	
CELLINA 5 B	GALATONE	26.85	0.01178	0.82438	1.03	5.16E+03	7.96E+03
CELLINA 5 M	GALATONE	25.73	0.02451	1.71	2.14	1.07E+04	
CELLINA 6 B	GALATONE	28.93	0.00296	0.20712	0.25890	1.30E+03	1.37E+03
CELLINA 6 M	GALATONE	28.76	0.00331	0.23143	0.28929	1.45E+03	
CELLINA7 B	GALATONE	27.72	0.00658	0.46072	0.57590	2.89E+03	3.17E+03
CELLINA 7 M	GALATONE	27.44	0.00789	0.69014	0.69014	3.46E+03	
LECCINO 1 B	GALATONE	0.00	0.00	0.00	0.00	0.00E+00	0.00E+00
LECCINO 1 M	GALATONE	0.00	0.00	0.00	0.00	0.00E+00	
LECCINO 2 B	GALATONE	30.14	0.00133	0.09293	0.11616	5.82E+02	2.91E+02
LECCINO 2 M	GALATONE	0.00	0.00	0.00	0.00	0.00E+00	
LECCINO 3 B	GALATONE	0.00	0.00	0.00	0.00	0.00E+00	0.00E+00
LECCINO 3 M	GALATONE	0.00	0.00	0.00	0.00	0.00E+00	
LECCINO 4 B	GALATONE	0.00	0.00	0.00	0.00	0.00E+00	2.26E+02
LECCINO 4 M	GALATONE	30.55	0.07208	0.09010	0.65475	4.52E+02	
LECCINO 5 B	GALATONE	27.44	0.00789	0.55220	0.69025	3.46E+03	1.73E+03
LECCINO 5 M	GALATONE	0.00	0.00	0.00	0.00	0.00E+00	
LECCINO 6 B	GALATONE	0.00	0.00	0.00	0.00	0.00E+00	0.00E+00
LECCINO 6 M	GALATONE	0.00	0.00	0.00	0.00	0.00E+00	
LECCINO 7 B	GALATONE	0.00	0.00	0.00	0.00	0.00E+00	0.00E+00
LECCINO7 M	GALATONE	0.00	0.00	0.00	0.00	0.00E+00	
OGLIAROLA 1 B	GALATONE	0.00	0.00	0.00	0.00	0.00E+00	8.54E+01 *[5]
OGLIAROLA 2 B	GALATONE	29.59	0.00190	0.13316	0.16645	8.34E+02	3.84E+03 *[6]
OGLIAROLA 3 B	GALATONE	36.65	0.00002	0.00124	0.00155	0.34E+02	3.89E+00 *[7]
OGLIAROLA 4 B	GALATONE	27.82	0.00616	0.43149	0.53937	2.70E+03	1.40E+03 *[8]

E=93.9%; r^2=0.981; Slope=-3.478

*[1] Mean with Ogliarola salentina 7B Cannole
*[2] Mean with Ogliarola salentina 8B Cannole
*[3] Mean with Ogliarola salentina 9B Cannole
*[4] Mean with Ogliarola salentina 10B Cannole
*[5] Mean with Ogliarola salentina 1M Galatone
*[6] Mean with Ogliarola salentina 2M Galatone
*[7] Mean with Ogliarola salentina 3M Galatone
*[8] Mean with Ogliarola salentina 4M Galatone

Cultivar	Plot	CT ratio	ng μL^{-1}	ng TOT	ng Tot g^{-1} sample	CFU g^{-1}	CFU value g^{-1} plant
OGLIAROLA 1 M	GALATONE	32.30	0.00039	0.02	0.02726	1.71E+02	8.54E+01 *[5]
OGLIAROLA 2 M	GALATONE	26.56	0.01563	1.09	1.36	6.85E+03	3.84E+03 *[6]
OGLIAROLA 3 M	GALATONE	0.00	0.00	0.00	0.00	0.00E+00	3.89E+00 *[7]
OGLIAROLA 4 M	GALATONE	33.17	0.00022	0.01537	0.01921	8.63E+01	1.40E+03 *[8]
OGLIAROLA 5 B	GALATONE	26.79	0.01354	0.94812	1.18	5.94E+03	5.04E+03
OGLIAROLA 5 M	GALATONE	27.35	0.00945	0.66129	0.82662	4.14E+03	
OGLIAROLA 6 B	GALATONE	30.99	0.00091	0.06353	0.07942	3.98E+02	2.35E+03
OGLIAROLA 6 M	GALATONE	27.28	0.00981	0.68700	0.85875	4.30E+03	
OGLIAROLA 7 B	GALATONE	28.06	0.00596	0.41731	0.52164	2.61E+03	2.91E+03
OGLIAROLA 7 M	GALATONE	27.75	0.00730	0.51130	0.639913	3.20E+03	

E=90.9%; r^2=0.991; Slope=-3.562

*[5] Mean with Ogliarola salentina 1B Galatone
*[6] Mean with Ogliarola salentina 2B Galatone
*[7] Mean with Ogliarola salentina 3B Galatone
*[8] Mean with Ogliarola salentina 4B Galatone

Table S3. The table reports the Ct value, X. *fastidiosa* gDNA ng µL^{-1} concentration, total X. *fastidiosa* gDNA amount, total X. *fastidiosa* gDNA amount per g sample, X. *fastidiosa* CFU equivalents per g sample and X. *fastidiosa* CFU equivalents per g plant sample. Each real time PCR run reports own standard curve parameters of efficiency (E), r^2 and slope. The data are reported considering October samplings (cultivars and plots are shown).

Cultivar	Plot	CT ratio	ng µL^{-1}	ng TOT	ng Tot g^{-1} sample	CFU g^{-1}	CFU value g^{-1} plant
CELLINA 1 B	CANNOLE	33.64	0.00003	0.00203	0.00254	1.27E+01	3.98E+02
CELLINA 1 M	CANNOLE	26.40	0.00346	0.24209	0.30261	1.52E+03	
CELLINA 2 B	CANNOLE	27.38	0.00178	0.12491	0.15614	7.83E+02	7.61E+02
CELLINA 2 M	CANNOLE	23.65	0.02186	1.53	1.91	9.59E+03	
CELLINA 3 B	CANNOLE	29.71	0.00038	0.02690	0.03362	1.69E+02	2.78E+02
CELLINA 3 M	CANNOLE	26.01	0.00446	0.31222	0.39027	1.96E+03	
CELLINA 4 B	CANNOLE	26.56	0.00308	0.21591	0.26989	1.35E+03	4.64E+02
CELLINA 4 M	CANNOLE	25.74	0.00542	0.37905	0.47382	2.37E+03	
CELLINA 5 B	CANNOLE	29.02	0.00060	0.04220	0.05275	2.64E+02	7.56E+02
CELLINA 5 M	CANNOLE	25.43	0.00681	0.47642	0.59553	2.98E+03	
CELLINA 6 B	CANNOLE	28.87	0.00066	0.04652	0.05816	2.91E+02	5.56E+03
CELLINA 6 M	CANNOLE	25.64	0.00542	0.39676	0.49595	2.49E+03	
CELLINA7 B	CANNOLE	27.51	0.00164	0.11484	0.14355	7.19E+02	2.17E+03
CELLINA 7 M	CANNOLE	23.88	0.01838	1.28	1.61	8.06E+03	
CELLINA 8 B	CANNOLE	29.41	0.00047	0.03325	0.041156	2.08E+02	1.49E+03
CELLINA 8 M	CANNOLE	0.00	0.00	0.00	0.00	0.00E+00	
CELLINA 9 B	CANNOLE	0.00	0.00	0.00	0.00	0.00E+00	0.00E+00
CELLINA 9 M	CANNOLE	0.00	0.00	0.00	0.00	0.00E+00	
CELLINA 10 B	CANNOLE	26.39	0.00345	0.24129	0.30161	1.51E+03	1.37E+02
CELLINA 10 M	CANNOLE	28.97	0.00062	0.04364	0.05455	2.73E+02	
OGLIAROLA 1 B	CANNOLE	31.85	0.00010	0.00700	0.00875	4.38E+01	8.08E+02
OGLIAROLA 1 M	CANNOLE	26.45	0.00359	0.25101	0.31376	1.57E+03	
OGLIAROLA 2 B	CANNOLE	26.60	0.00302	0.21144	0.26430	1.32E+03	1.09E+04
OGLIAROLA 2 M	CANNOLE	22.47	0.04656	3.25	4.07	2.04E+04	
OGLIAROLA 3 B	CANNOLE	28.31	0.00098	0.06855	0.08569	4.29E+02	6.17E+02
OGLIAROLA 3 M	CANNOLE	27.34	0.00183	0.12832	0.16040	8.04E+02	
OGLIAROLA 4 B	CANNOLE	26.09	0.0445	0.31150	0.38938	1.95E+03	1.18E+03
OGLIAROLA 4 M	CANNOLE	28.48	0.00092	0.06444	0.08055	4.04E+02	
OGLIAROLA 5 B	CANNOLE	30.43	0.00026	0.01799	0.02249	1.13E+02	9.14E+02
OGLIAROLA 5 M	CANNOLE	26.20	0.00391	0.27390	0.34238	1.72E+03	
OGLIAROLA 6 B	CANNOLE	29.22	0.00054	0.03754	0.04692	2.35E+02	2.13E+02 *1
OGLIAROLA 7 B	CANNOLE	29.11	0.00057	0.03999	0.04999	2.51E+02	7.92E+02 *2
OGLIAROLA 8 B	CANNOLE	26.23	0.00384	0.26882	0.33602	1.68E+03	3.04E+03 *3
OGLIAROLA 9 B	CANNOLE	27.98	0.00122	0.08535	0.10668	5.35E+02	7.36E+02 *4
OGLIAROLA 10 B	CANNOLE	26.16	0.00401	0.28087	0.35109	1.76E+03	8.88E+02*5

E=94.2%; r^2=0.996; Slope=-3.471

*1 Mean with Ogliarola salentina 6M Cannole

*2 Mean with Ogliarola salentina 7M Cannole

*3 Mean with Ogliarola salentina 8M Cannole

*4 Mean with Ogliarola salentina 9M Cannole

*5 Mean with Ogliarola salentina 10M Cannole

Cultivar	Plot	CT ratio	ng μL^{-1}	ng TOT	ng Tot g^{-1} sample	CFU g^{-1}	CFU value g^{-1} plant
OGLIAROLA 6 M	CANNOLE	31.45	0.00044	0.03050	0.03813	1.91E+02	2.13E+02 *[1]
OGLIAROLA 7 M	CANNOLE	28.30	0.00304	0.21284	0.26605	1.33E+03	7.92E+02 *[2]
OGLIAROLA 8 M	CANNOLE	26.37	0.01001	0.70077	0.87596	4.39E+03	3.04E+03 *[3]
OGLIAROLA 9 M	CANNOLE	28.88	0.00214	0.14946	0.18683	9.36E+02	7.36E+02 *[4]
OGLIAROLA 10 M	CANNOLE	35.46	0.00004	0.00259	0.00323	1.62E+01	8.88E+02 *[5]
CELLINA 1 B	GALATONE	26.34	0.01020	0.71383	0.89229	4.47E+03	5.66E+03
CELLINA 1 M	GALATONE	25.66	0.01561	1.09	1.36	6.84E+03	
CELLINA 2 B	GALATONE	25.17	0.02092	1.46	1.83	9.18E+03	9.68E+03
CELLINA 2 M	GALATONE	25.00	0.02320	1.62	2.03	1.02E+04	
CELLINA 3 B	GALATONE	29.45	0.00151	0.10551	0.13188	6.61E+02	1.37E+03
CELLINA 3 M	GALATONE	27.59	0.00472	0.33032	0.41290	2.07E+03	
CELLINA 4 B	GALATONE	25.63	0.01574	1.10	1.37	6.90E+03	5.39E+03
CELLINA 4 M	GALATONE	26.17	0.01131	0.79194	0.98993	4.96E+03	
CELLINA 5 B	GALATONE	31.45	0.00621	0.43439	0.54299	2.72E+03	5.91E+03
CELLINA 5 M	GALATONE	25.18	0.02077	1.45	1.81	9.11E+03	
CELLINA 6 B	GALATONE	26.82	0.00763	0.53399	0.66749	3.35E+03	1.71E+03
CELLINA 6 M	GALATONE	32.90	0.00018	0.01252	0.01565	7.85E+01	
CELLINA7 B	GALATONE	26.55	0.00895	0.62680	0.78350	3.93E+03	2.29E+02
CELLINA 7 M	GALATONE	29.44	0.00151	0.10562	0.13203	6.62E+02	
LECCINO 1 B	GALATONE	26.38	0.00996	0.69702	0.87128	4.37E+03	2.19E+03 *[6]
LECCINO 2 B	GALATONE	0.00	0.00	0.00	0.00	0.00E+00	0.00E+00 *[7]
LECCINO 3 B	GALATONE	29.58	0.00139	0.09698	0.12122	6.08E+02	3.04E+02 *[8]
LECCINO 4 B	GALATONE	0.00	0.00	0.00	0.00	0.00E+00	0.00E+00 *[9]
LECCINO 5 B	GALATONE	0.00	0.00	0.00	0.00	0.00E+00	1.18E+00 *[10]
LECCINO 6 B	GALATONE	27.45	0.00516	0.36134	0.45168	2.26E+03	1.13E+03 *[11]

E=90%; r^2=0.996; Slope=-3.589

*[1] Mean with Ogliarola salentina 6B Cannole
*[2] Mean with Ogliarola salentina 7B Cannole
*[3] Mean with Ogliarola salentina 8B Cannole
*[4] Mean with Ogliarola salentina 9B Cannole
*[5] Mean with Ogliarola salentina 10B Cannole
*[6] Mean with Leccino 1M Cannole
*[7] Mean with Leccino 2M Cannole
*[8] Mean with Leccino 3M Cannole
*[9] Mean with Leccino 4M Cannole
*[10] Mean with Leccino 5M Cannole
*[11]Mean with Leccino 6M Cannole

Cultivar	Plot	CT ratio	ng μL^{-1}	ng TOT	ng Tot g^{-1} sample	CFU g^{-1}	CFU value g^{-1} plant
LECCINO 1 M	GALATONE	33.70	0.00002	0.00138	0.00172	8.63E+00	2.19E+03 *[6]
LECCINO 2 M	GALATONE	0.00	0.00	0.00	0.00	0.00E+00	0.00E+00 *[7]
LECCINO 3 M	GALATONE	0.00	0.00	0.00	0.00	0.00E+00	3.04E+02 *[8]
LECCINO 4 M	GALATONE	0.00	0.00	0.00	0.00	0.00E+00	0.00E+00 *[9]
LECCINO 5 M	GALATONE	35.29	0.00001	0.00038	0.00047	2.37E+00	1.18E+00 *[10]
LECCINO 6 M	GALATONE	0.00	0.00	0.00	0.00	0.00E+00	1.13E+03 *[11]
LECCINO 7 B	GALATONE	28.76	0.00061	0.04277	0.05346	2.68E+02	2.75E+02
LECCINO7 M	GALATONE	33.83	0.00002	0.00110	0.00137	6.89E+00	
OGLIAROLA 1 B	GALATONE	25.07	0.00842	0.58957	0.73697	3.69E+03	2.06E+03
OGLIAROLA 1 M	GALATONE	28.10	0.00095	0.06665	0.08332	4.18E+02	
OGLIAROLA 2 B	GALATONE	26.48	0.00307	0.21463	0.26829	1.34E+03	4.11E+03
OGLIAROLA 2 M	GALATONE	24.20	0.01570	1.09	1.37	6.88E+03	
OGLIAROLA 3 B	GALATONE	32.91	0.00003	0.00236	0.00295	1.48E+01	5.04E+01
OGLIAROLA 3 M	GALATONE	30.29	0.00020	0.01374	0.01718	8.61E+01	
OGLIAROLA 4 B	GALATONE	23.92	0.01931	1.35	1.68	8.47E+03	6.89E+03
OGLIAROLA 4 M	GALATONE	24.58	0.01213	0.84910	1.06	5.32E+03	
OGLIAROLA 5 B	GALATONE	23.43	0.02737	1.91	2.39	1.20E+04	6.84E+03
OGLIAROLA 5 M	GALATONE	26.17	0.00381	0.26655	0.33318	1.67E+03	
OGLIAROLA 6 B	GALATONE	28.29	0.00083	0.05825	0.07281	3.65E+02	3.31E+02
OGLIAROLA 6 M	GALATONE	28.58	0.00068	0.04726	0.05908	2.96E+02	
OGLIAROLA 7 B	GALATONE	30.71	0.00019	0.01348	0.01685	8.45E+01	2.25E+03
OGLIAROLA 7 M	GALATONE	24.82	0.01007	0.70514	0.88142	4.42E+03	

E=105%; r^2=0.995; Slope=-3.201

*[6] Mean with Leccino 1B Cannole

*[7] Mean with Leccino 2B Cannole

*[8] Mean with Leccino 3B Cannole

*[9] Mean with Leccino 4B Cannole

*[10] Mean with Leccino 5B Cannole

*[11]Mean with Leccino 6B Cannole
